# Heterovalent Substitution of K_2_SrP_2_O_7_:Cr^3+^ to Achieve Anti-Thermal-Quenching Broadband Near-Infrared Luminescence

**DOI:** 10.1021/acsaom.5c00201

**Published:** 2025-08-11

**Authors:** Hexi Zhang, Yuanbing Mao

**Affiliations:** Department of Chemistry, 146315 Illinois Institute of Technology, Chicago, Illinois 60616, United States

**Keywords:** Heterovalent Substitution, Near-Infrared Luminescence, Anti-Thermal-Quenching, K_2_SrP_2_O_7_, Cr^3+^

## Abstract

Broadband near-infrared (NIR) light sources based on phosphor-converted light-emitting diodes are highly desirable for biochemical analysis and medical diagnosis applications. However, thermal quenching remains a demanding challenge for developing efficient NIR phosphors. Herein, we report the enhancement of both quantum efficiency and thermal stability in Cr^3+^-activated K_2_SrP_2_O_7_ phosphors through a heterovalent substitution strategy by replacing Sr^2+^ with Al^3+^ in K_2_Sr_1–*x*_Al_*x*_P_2_O_7_ (0.05 ≤ *x* ≤ 0.2) to obtain optimized broadband NIR emission. Structural modulation via Al^3+^ substitution leads to the optimized composition, K_2_Sr_0.88_Al_0.1_P_2_O_7_:0.02Cr^3+^, which emits across a broad NIR range of 650–1100 nm peaking at 807 nm with a full width at half-maximum of ∼130 nm under 448 nm excitation. Remarkably, its emission intensity at 150 °C remains 120% of the initial value at room temperature, demonstrating a rare antithermal-quenching behavior. Temperature-dependent XRD studies further reveal that Al^3+^ substitution effectively suppresses lattice expansion at elevated temperatures, indicating enhanced lattice stability under thermal excitation. Detailed structural and spectral analyses show that the substitution enhances local site symmetry, reduces electron–phonon coupling, increases thermally induced absorption probability, and fortifies energetic barriers against nonradiative transitions. These synergistic effects collectively endow this NIR phosphor with a superior thermal stability. Furthermore, NIR light-emitting diodes fabricated with this phosphor exhibit strong potential for applications in information identification, nondestructive detection, and night vision technologies. This study demonstrates a local structure engineering strategy for designing thermally robust Cr^3+^-activated NIR phosphors, offering valuable insights into material discovery and NIR spectroscopy device development.

## Graphical abstract

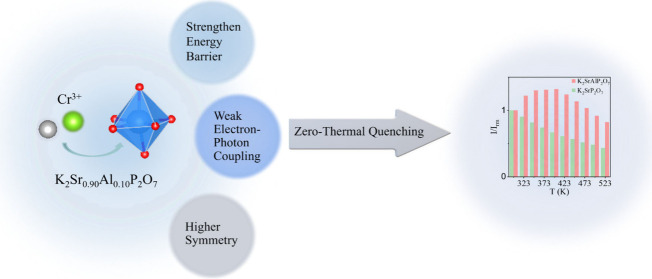

## Introduction

Near-infrared (NIR) spectroscopy technology has attracted extensive attention for being utilized across a wide array of fields: food processing, night vision, agriculture, biosensing, etc.^1–4^ The interest in NIR light arises from its numerous advantages, such as its invisibility to the human eye, strong penetrating ability, and characteristic absorption by specific molecules. Traditional solid-state NIR light-emitting devices, such as tungsten filament lamps and halogen lamps, are limited from large size, low efficiency, and high working temperature.^5,6^ On the contrary, phosphor-converted light-emitting diodes (pc-LEDs) based on UV or blue LED chips and optimized NIR phosphors are candidates for future NIR light sources as a result of their environmental friendliness, low cost, and long life stability.^7^ The world’s first broadband NIR pc-LED was produced by OSRAM in 2016; massive progress has been accomplished in NIR phosphors that can be stimulated by commercial blue LED chips.^8^ To date, the operational temperature of commercial high-power NIR LED chips can reach up to about 150–200 °C.^9^ Furthermore, it has been observed that the majority of NIR phosphors experience a loss of emission intensity exceeding 20% upon exposure to temperatures elevated to 150 °C, indicating inadequate thermal stability.^10^ These limitations are significantly below the desired performance criteria for NIR pc-LED devices, underscoring a critical area for improvement.

Among various transition metals, rare earth ions, and main group elements explored for NIR emissions, Cr^3+^ impressively is one of the most widely used activators.^11–13^ It possesses tunable broadband NIR emissions and its broad excitation bands, which match well with the emissions of a cost-effective commercial blue diode chip. However, despite its advantages, many Cr^3+^-activated phosphors that exhibit broadband emission with peak wavelengths >800 nm suffer from poor thermal stability, which limits their practical applications. Given that pc-LED devices can reach temperatures as high as 150 °C due to energy loss during blue-to-NIR light conversion, enhancing the thermal resistance of Cr^3+^-doped phosphors remains a crucial challenge.

Several strategies have been proposed to strengthen thermal stability of NIR phosphors, including crystallinity enhancement, heterovalent substitution, defect engineering, utilizing glass-ceramic phosphors, etc.^13–15^ Among these approaches, heterovalent substitution at cationic and anionic sites in solid-state materials has emerged as a highly effective strategy. This approach modifies the chemical composition of host lattices by introducing high levels of cation disorder, directly influencing the local lattice environments surrounding activator ions, thereby optimizing electron-photon interactions, improving luminescent efficiency and enhancing thermal stability.^16^ For example, Tang et al. demonstrated that Lu^3+^ substitution in enhanced thermal stability, with K_2_Hf_0.8_Lu_0.2_Si_3_O_9_:Eu^2+^ retaining 94% of its PL intensity at 200 °C, compared to 69% for the unsubstituted counterpart.^17^ Similarly, Jiang et al. reported that replacing Zn^2+^ with Ga^3+^ in ZnGa_2_O_4_:Cr^3+^improved both emission intensity and thermal stability, attributed to defect level formation. The optimized Zn_0.98_Ga_2.02_O_4+δ_:Cr^3+^ maintained 77.3% of its emission at 423 K, compared to 73.8% for the unsubstituted counterpart.^18^ In our recent work, we demonstrated that heterovalent substitution in K_2_CaP_2_O_7_:Cr^3+^ not only stabilized the Cr^3+^ emitter but also generated oxygen vacancy defects, which significantly improved thermal quenching properties at elevated temperatures.^19^ These findings established heterovalent substitution as a versatile strategy for modulating host lattices environment, mitigating nonradiative losses, and optimizing luminescent performance.

In this work, we explored heterovalent substitution design strategies to further enhance the performance of the NIR phosphors. We developed a K_2_Sr_0.88_Al_0.1_P_2_O_7_:0.02Cr^3+^ phosphor through heterovalent substitution to achieve structural modulations toward enhanced quantum efficiency and thermal stability. This phosphor exhibited broad NIR emission ranging from 650 to 1100 nm with a peak centered at 807 nm and a large full width at half-maximum (fwhm) of about 130 nm under excitation at 448 nm. It was found that both the internal quantum efficiency (IQE) and thermal stability of this Cr^3+^-activated K_2_SrP_2_O_7_ phosphor could be improved via chemical substitution of Sr^2+^ by Al^3+^ in the K_2_SrP_2_O_7_ host. Structural and spectral analyses suggest that enhanced thermal stability in the K_2_Sr_0.9_Al_0.1_P_2_O_7_ host may arise from increased [Sr/AlO_6_] octahedral symmetry, a strengthened energy barrier, and ultraweak EPC effect. Furthermore, an optimized NIR phosphor-based pc-LED light source was fabricated and demonstrated great potential in information identification, nondestructive detection, and night vision technologies.

## Experimental Section

### Synthesis

Powder samples of K_2_Sr_0.99‑*x*_Al_*x*_P_2_O_7_:0.01Cr^3+^ (*x* = 0, 0.05, 0.1, 0.15 and 0.2) and K_2_Sr_0.90‑*y*_Al_0.1_P_2_O_7_:*y*Cr^3+^ (*y* = 0.005, 0.01, 0.02 and 0.03) were prepared via a solid-state reaction. Starting materials of K_2_CO_3_ (Sigma-Aldrich, 99.0%), SrCO_3_ (Sigma-Aldrich, 99.9%), NH_4_H_2_PO_4_ (Acros Organics, 99.9%), and Cr­(NO_3_)_3_·9H_2_O (Acros Organics, 99%) were weighed according to stoichiometric ratios. The samples were initially ground thoroughly for 1 h, after which the powder mixtures were compacted into pellets using a uniaxial press provided by Vivtek. The pellets were then presintered at 300 °C for 1 h, followed by sintering at 800 °C for 5 h twice while cooling down to room temperature in between. The pellets were subsequently crushed and ground into a fine powder, which was then used for further analysis.

### LED Fabrication

The K_2_Sr_0.88_Al_0.1_P_2_O_7_:0.02Cr^3+^ NIR-emitting phosphor was first mixed with epoxy resins A and B in a 1:1 mass ratio and then coated onto ∼ 450 nm blue chips. The mixture was cured at 80 °C for 20 min, forming the final LED devices.

### Characterization

X-ray powder diffraction (XRD) patterns were performed on a benchtop XRD device (Bruker D2 Phaser) with Cu Kα radiation (λ = 1.5406 Å) at 45 kV and 40 mA. The process for phase identification and quantitation was facilitated by the software PDF-4. The crystallographic data underwent analysis via Rietveld refinements, employing the GSAS-II software.^20^ Both elemental mapping and morphology images were obtained by utilizing energy-dispersive spectroscopy (EDS) on the field emission scanning electron microscope (FESEM) instrument (JEOL, JSMF6701). Absorption spectra of each bulk sample material were captured by using a Cary 5000 UV–vis-NIR double beam spectrophotometer with a monochromator. The UV–vis diffuse reflectance spectra was measured using a PerkinElmer Lambda 950 UV/vis/NIR spectrometer. Temperature-dependent PXRD analysis patterns were taken on a STOE STADI-MP instrument equipped with Ag Kα1 and Mo Kα1 radiation sources. Optical measurements including photoluminescence (PL) and PL excitation (PLE) spectra were realized using a computer-controlled spectrofluorometer (FLS 1000 spectrofluorometer, Edinburgh Instruments Ltd.) equipped with a 450 W xenon arc lamp as an excitation source and a Hamamatsu R928P photomultiplier tube (photocounting mode in 180–950 nm) as an emission detector. The low-temperature heat capacity was determined by using a Quantum Design Physical Properties Measurement System (PPMS). To determine luminescence thermometric performance, temperature-dependent luminescence, including PL spectra and lifetime, was measured by loading the powder samples on an exterior Linkam heating/cooling stage (controllable temperature at 78–873 K) equipped with the FLS 1000 spectrofluorometer. Emission/excitation fiber optics were used to convey luminescence signals from the stage to the chamber of the spectrofluorometer.

## Results and Discussion

### Phase Analysis of K_2_Sr_0.99‑x_Al_*x*_P_2_O_7_:0.01Cr^3+^

The phase purity and crystallinity of the as-prepared K_2_Sr_0.99‑*x*_Al_*x*_P_2_O_7_:0.01Cr^3+^ (0.05 ≤ *x* ≤ 0.2) phosphors were monitored by X-ray diffraction (XRD), and the results are demonstrated in Figure 1a. All peaks were indexed using a monoclinic cell (*P*2_1_/*c*) with parameters close to K_2_SrP_2_O_7_ (PDF# 01-077-0727), and the proper introduction of Al^3+^ does not disrupt the phase purity. The unit cell of K_2_SrP_2_O_7_ is built by the Sr^2+^ octahedral connected with the tetrahedral PO_4_ groups on the corners and the K^+^ ions located between them.^21,22^ It is evident that the diffraction peaks shift toward a higher angle in the enlarged pattern (2θ = 29–29.9°), illustrating the substitution of Al^3+^ ions for Sr^2+^ due to the smaller ionic radius of Al^3+^ (0.535 Å, CN = 6) compared to that of Sr^2+^ (1.18 Å, CN = 6). The observed shift of diffraction peaks toward higher angles in the enlarged XRD pattern in the 2θ range 29–29.9° suggests the successful incorporation of Al^3+^ into the Sr^2+^ sites. This shift is attributed to the smaller ionic radius of Al^3+^ (0.535 Å, CN = 6) compared to Sr^2+^ (1.18 Å, CN = 6), leading to lattice contraction. Such heterovalent substitution aligns with well-established phosphor design strategies, where Al^3+^ replacing larger divalent cations induces lattice contraction and corresponding XRD peak shifts, as reported in analogous systems of α-Na_4_Zn­(PO_4_)_2_ by partially substituting Zn^2+^ with Ga^3+^ and Al^3+^, copper­(I)-doped 0D cesium zinc halide by codoping with Al^3+^ at Zn sites, and Ca_9_LiMg­(PO_4_)_7_:Eu^2+^ by heterovalent substitution of Al^3+^ for Mg^2+^.^23–25^ Given that the crystal field stabilization energy of Cr^3+^ ions in octahedrons is eight times higher than that in tetrahedrons, the [SrO_6_] octahedra are suitable for Cr^3+^ to occupy.^26^ All the diffraction peaks of the XRD patterns of the K_2_Sr_0.9‑y_Al_0.1_P_2_O_7_:yCr^3+^ (y = 0.005, 0.01, 0.02, and 0.03) phosphors are well-indexed to the standard K_2_SrP_2_O_7_ phase (Figure S1a), confirming their phase purity.

**Figure 1:**
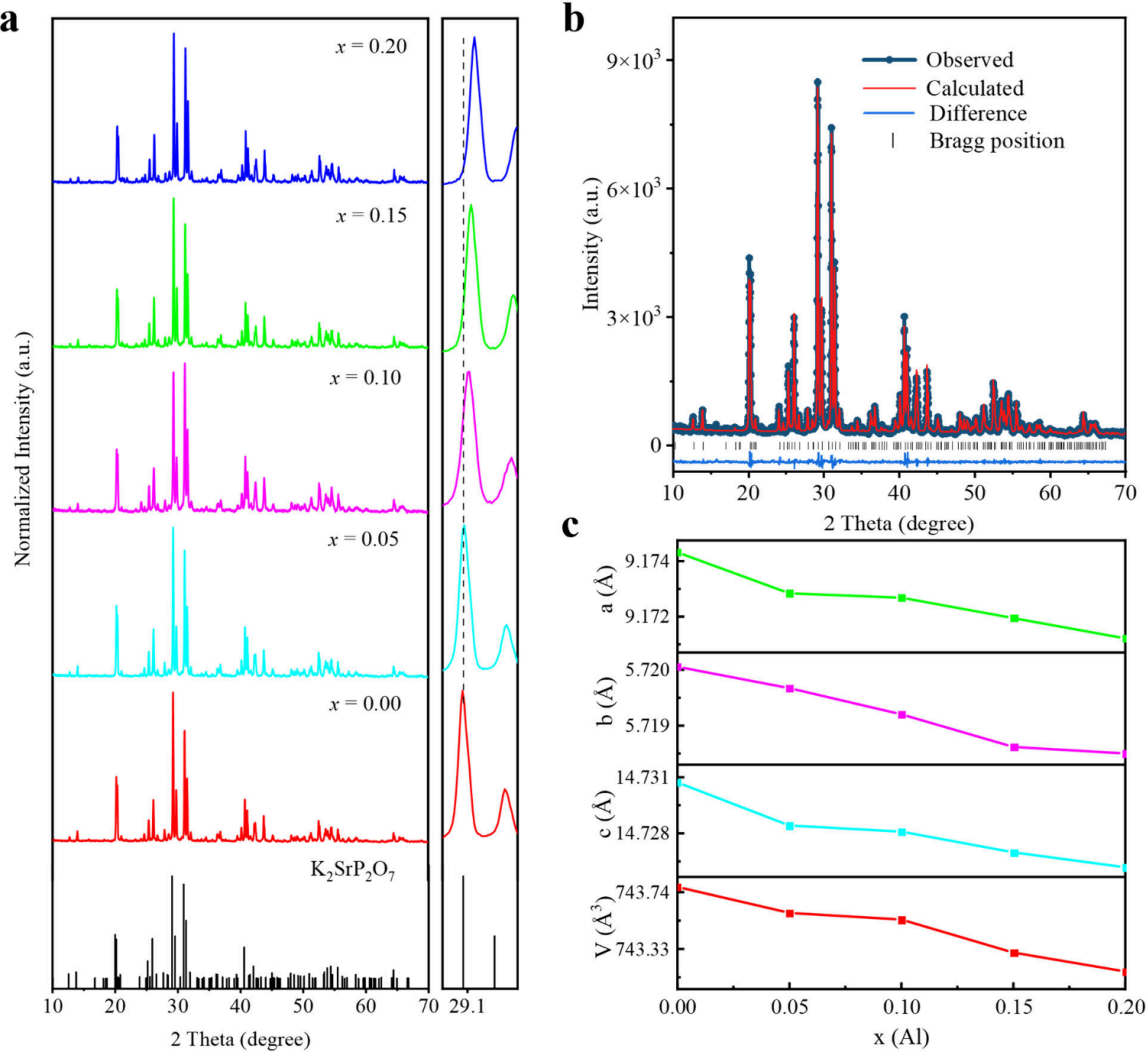
(a) XRD patterns of K_2_Sr_0.99‑*x*_Al_*x*_P_2_O_7_:0.01Cr^3+^ (0.05 ≤ *x* ≤ 0.2) and the standard pattern of PDF card No. 01-077-0727 of K_2_SrP_2_O_7_ as a reference. The magnified selected diffraction peaks at ∼ 29.1° are shown in the right. (b) Rietveld refinement result of K_2_Sr_0.88_Al_0.1_P_2_O_7_:0.02Cr^3+^. (c) Refined lattice parameters of *a*, *b*, *c*, and *V* of K_2_Sr_0.99‑*x*_Al_*x*_P_2_O_7_:0.01Cr^3+^ as a function of the substitution ratio of *x*(Al).

To investigate the detailed structural information on K_2_Sr_0.99‑*x*_Al_*x*_P_2_O_7_:0.01Cr^3+^ (0.05 ≤ *x* ≤ 0.2) phosphors, the K_2_Sr_0.88_Al_0.1_P_2_O_7_:0.02Cr^3+^ structure was taken as a starting model for Rietveld refinement as shown in Figure 1b, which was performed using GSAS-II software.^20^ The refined results are provided in Table S1. The small residual factors (*R*_*wp*_ = 7.14%) imply that the refinement results are acceptable. With the increase in Al^3+^ content in K_2_Sr_0.99‑*x*_Al_*x*_P_2_O_7_:0.01Cr^3+^, the refined cell parameters *a*, *b*, *c*, and *V* decrease gradually (Figure 1c), which emphasizes the replacement of Sr^2+^ sites by smaller Al^3+^ ions.

The scanning electron microscopy (SEM) and elemental mapping images of the selected K_2_Sr_0.88_Al_0.1_P_2_O_7_:0.02Cr^3+^ sample are shown in Figure S2. The individual particles of this sample are irregular in shape. X-ray energy-dispersive spectroscopy (EDS) mapping on a randomly selected single particle showed that the detected K, Sr, Al, P, O, and Cr elements as initially loaded were uniformly distributed on the particle.

### Photoluminescence Properties

PLE and PL spectra of our K_2_Sr_0.99‑*x*_Al_*x*_P_2_O_7_:0.01Cr^3+^ (0.05 ≤ *x* ≤ 0.2) phosphors at room temperature (RT) were recorded in Figure 2a. Under 448 nm excitation, the phosphor exhibits a broadband emission peak ranging from 650 to 1100 nm, with a peak between 785 and 807 nm and a fwhm of about 130 nm, which should be attributed to the spin-allowed ^4^T_2_ → ^4^A_2_ (^4^F) transition of Cr^3+^ ions in octahedral coordination as mentioned above. Figure S3 shows the absorption and diffuse reflectance spectra of K_2_SrP_2_O_7_ and K_2_Sr_0.88_Al_0.1_P_2_O_7_:0.02Cr^3+^. As shown, the K_2_SrP_2_O_7_ host produces almost no absorption in the visible spectrum while the absorption spectrum of the K_2_Sr_0.88_Al_0.1_P_2_O_7_:0.02Cr^3+^ sample exhibits two strong absorption bands in the region of 400–500 nm (blue) and 550–750 nm (red), which derive from the ^4^A_2_ → ^4^T_1_ and ^4^A_2_ → ^4^T_2_ transitions of Cr^3+^, respectively. PLE spectra of the K_2_Sr_0.99‑*x*_Al_*x*_P_2_O_7_:0.01Cr^3+^ (0.05 ≤ *x* ≤ 0.2) phosphors also give consistent results with absorption spectra. As shown in Figure 2b, with increasing *x*(Al), PL emission peaks of the K_2_Sr_0.99‑*x*_Al_*x*_P_2_O_7_:0.01Cr^3+^ phosphors initially exhibit a blue shift, followed by a slightly red shift. Meanwhile, the luminescence intensity first increased and then decreased, reaching an optimal value at *x*(Al) = 0.1. This trend suggests that nonradiative relaxation of Cr^3+^ emitters in the K_2_Sr_0.90_Al_0.1_P_2_O_7_ samples may be suppressed, which will be discussed later. Heterovalent substitution of trivalent cations for divalent cations has been widely studied in various phosphor systems, such as Cs_3_(Zn_1–*x*_Al_*x*_)­Cl_5_:0.05Cu^+^, Ca_9_LiMg_1–*x*_Al_2*x*/3_(PO_4_)_7_:0.1Eu^2+^, and Ca_3_Si_2–*x*_M_*x*_O_7_:Eu^3+^ (M = Al, P), as it induces local structural modulations, charge compensation effects, and defect engineering, which can enhance luminescence properties.^24,25,27^ The observed spectral shifts and intensity variations in this study indicate that Al^3+^ incorporation influences the local coordination environment of Cr^3+^ and modifies the electronic structure of the host lattice.

**Figure 2:**
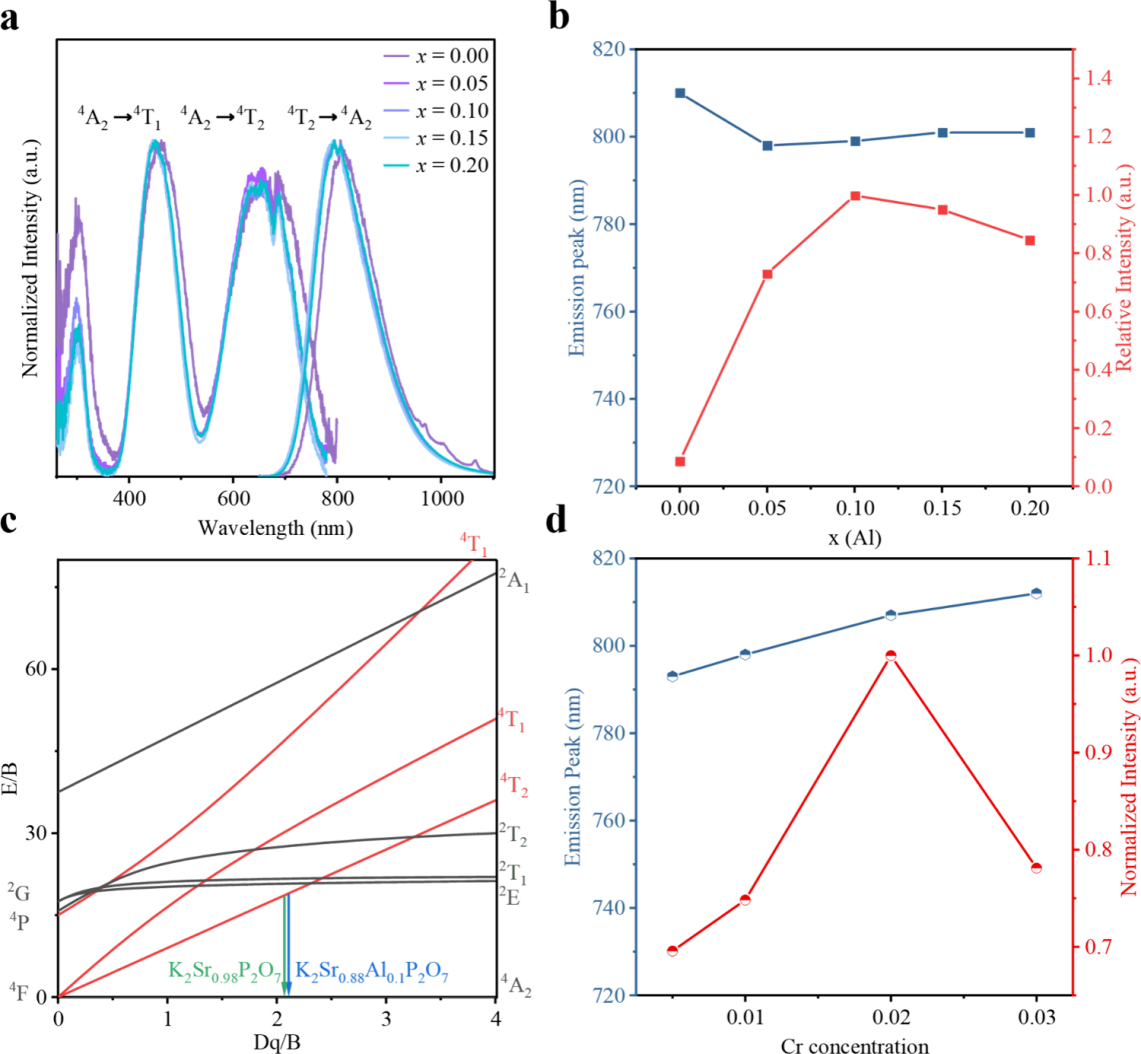
(a) Normalized PLE (λ_em_ = 810, 785, 799, 801, and 801 nm with *x* = 0, 0.05, 0.1, 0.15, and 0.2, respectively) and PL (λ_ex_ = 448 nm) spectra of the K_2_Sr_0.99‑*x*_Al_*x*_P_2_O_7_:0.01Cr^3+^ (0 ≤ *x* ≤ 0.2) phosphors. (b) The broadband NIR emission peak position and the relevant relative NIR emission peak intensity of the K_2_Sr_0.99‑*x*_Al_*x*_P_2_O_7_:0.01Cr^3+^ (*x* = 0–0.2) phosphors at λ_em_ = 810, 785, 799, 801, and 801 nm with *x* = 0, 0.05, 0.1, 0.15, and 0.2, respectively. (c) Tanabe–Sugano energy level diagram of Cr^3+^ in the K_2_Sr_0.98_P_2_O_7_:0.02Cr^3+^ and K_2_Sr_0.88_Al_0.1_P_2_O_7_:0.02Cr^3+^ phosphors. (d) Broadband emission peak and normalized NIR emission intensity of the K_2_Sr_0.9‑*y*_Al_0.1_P_2_O_7_:*y*Cr^3+^ (*y* = 0.005, 0.01, 0.02 and 0.03) phosphors as a function of Cr doping concentration at λ_em_ = 793, 798, 807, and 812 nm with *y* = 0.005, 0.01, 0.02, and 0.03, respectively) under λ_ex_ = 448 nm.

These findings underscore the influence of Sr^2+^ ↔ Al^3+^ substitution on the local crystal structure and optical properties of the K_2_SrP_2_O_7_:0.01Cr^3+^ phosphor. Additionally, the excitation spectra recorded at different emission spectral positions of the K_2_Sr_0.88_Al_0.1_P_2_O_7_:0.02Cr^3+^ sample (Figure S4) reveal nearly identical profiles, indicating that the emission originates from a single type of luminescent center. This is consistent with structural considerations: the ionic radius of Cr^3+^ (0.615 Å, CN = 6) more closely matches that of Sr^2+^ (1.18 Å, CN = 6) than K^+^ (1.55 Å, CN = 9), and Cr^3+^ ions typically favor octahedral coordination. These results further confirm that Cr^3+^ ions are occupying the Sr^2+^ lattice sites. On the basis of the Kubelka–Munk function,^28^ the optical band gap of the K_2_SrP_2_O_7_ and K_2_Sr_0.9_Al_0.1_P_2_O_7_ host was calculated to be 4.07 and 4.11 eV, respectively (Figure S3). This increase in the band gap upon the introduction of Al^3+^ suggests that the energy difference between the conduction band and the lowest excited state is widened. As a result, the likelihood of Cr^3+^ ionization is reduced, and the transition of excited-state electrons to the conduction band becomes more difficult, thereby enhancing the luminescence intensity.^29^ Additionally, the absorption spectra also indicate that the absorption region of our K_2_Sr_0.88_Al_0.1_P_2_O_7_:0.02Cr^3+^ phosphor is broad and matches well with that of the efficient blue chips.

Considering the absence of external shell electron shielding in Cr^3+^ ions, there exists a pronounced interaction with the crystal field and lattice vibrations. This interaction is attributable to the spatial extension of the *d* electron wave functions within crystals. Consequently, this phenomenon facilitates the manifestation of diverse optical properties in phosphors doped with Cr^3+^ ions.^11^ When considering Cr^3+^ ions in the center of coordinated octahedrons (*O*_h_ symmetry), the influence of the host lattice on luminescence properties is expressed by certain spectroscopic parameters of *D*_q_ and *B*, which can be roughly estimated by the following equations:^30^

1 10Dq=E(T42)

2 $$\frac{Dq}{B}=\frac{15\left(m-8\right)}{\left(m^{2}-10m\right)}$$

3 $$m=\frac{\Delta E}{Dq}$$

4 ΔE=E(T41)−E(T42)

where *D*_q_ represents the crystal field parameter and *B* is the Racah parameter. *E*(^4^T_1_) and *E*(^4^T_2_) are the equilibrium positions of the ^4^T_1_ and ^4^T_2_ levels obtained from the excitation spectrum. Thus, as shown in Figure 2c, *D*_q_, *B*, and *D*_q_/*B* values of the K_2_Sr_0.88_Al_0.1_P_2_O_7_:0.02Cr^3+^ phosphor are calculated to be 1520 cm^–1^, 722 cm^–1^, and 2.11, respectively. Those values of the K_2_Sr_0.98_P_2_O_7_:0.02Cr^3+^ counterpart were 1486 cm^–1^, 716 cm^–1^, and 2.08, respectively, which indicates that the crystal field strengthened and the Cr^3+^ indeed entered a weak crystal field (*D*_q_/*B* < 2.3) so that a broad emission appeared upon the heterovalent substitution.^26^ *D*_q_/*B* values of our K_2_Sr_0.99‑*x*_Al_*x*_P_2_O_7_:0.01Cr^3+^ (0.05 ≤ *x* ≤ 0.2) phosphors are calculated and listed in Table S3. These findings align with prior studies on the effects of smaller cations that replace larger cations in phosphor systems, such as Gd_2–*x*_Al_*x*_GaSbO_7_:Cr^3+^, CaSc_1–*x*_Al_1+*x*_SiO_6_:Cr^3+^, and Sr_1–*x*_La_1+*x*_ZnO_3.5+*x*/2_:Bi^3+^ (0 ≤ *x* ≤ 0.4), highlighting the role of local structural adjustments in modulating the electronic environments of Cr^3+^.^29,31,32^

Figure 2d shows the integrated emission intensity and emission peak position of the K_2_Sr_0.9‑*y*_Al_0.1_P_2_O_7_:*y*Cr^3+^ (*y* = 0.005, 0.01, 0.02, and 0.03) phosphors under 448 nm excitation. The optimized doping concentration of Cr^3+^ was determined to be 2 mol %, beyond which concentration quenching started to occur due to the increasing probability of nonradiative transitions between Cr^3+^ ions (Figure S5). However, the emission peak positions with different Cr^3+^ concentrations present a red shift (∼12 nm) with the increase of the Cr^3+^ concentration. This red-shift may arise from energy transfer among Cr^3+^ ions including phonon-assisted relaxation or migration to lower-energy sites. Similar trends have been reported in other Cr^3+^-doped phosphor systems, such as LiScO_2_:Cr^3+^.^33^ The IQE of our K_2_Sr_0.98‑*x*_Al_*x*_P_2_O_7_:0.02Cr^3+^ (*x* = 0 and 0.1) phosphors is determined to be 14.6% and 40.1%, respectively (Figure S6). The corresponding external quantum efficiencies and absorbance values were 10.3% and 70.6% for *x* = 0, and 35.2% and 87.7% for *x* = 0.1, respectively, demonstrating a significant enhancement upon Al^3+^ substitution.

Accordingly, the variation of the local structural distortion of the [Sr/AlO_6_] octahedron depending on Sr^2+^ ↔ Al^3+^ substitution has been discussed to evaluate the structure–property correlation, and it can be described by the following equation:^34^

5 $$D_{dis}=\frac{1}{n}\overset{i=1}{\underset{n}{\sum }}\frac{\left|d_{i}-d_{av}\right|}{d_{av}}$$

where *D*_dis_ represents the lattice distortion, *n* is the coordination number, *d* means the distance from Sr to the *i*th coordinating O atoms, and *d*_i_ is the average Sr–O distance as shown in Table S2. The derived *D*_dis_ value of [Sr/AlO_6_] demonstrates a reduction from 3.9% (*x* = 0) to 2.3% (*x* = 0.1), as shown in Figure S7, indicative of an increased structural symmetry with an increasing Al^3+^ substitution of Sr^2+^.

Furthermore, it is well-established that the shortened decay time of an emitting center typifies a distorted environment, while elongated decay values and enhanced thermal resistance are characteristic of a symmetrical site.^35,36^ Therefore, the PL decay curves of the K_2_Sr_0.98‑*x*_Al_*x*_P_2_O_7_:0.02Cr^3+^ (*x* = 0 and 0.1) phosphors monitored at 807 under 448 nm excitation were recorded at RT as shown in Figure S8. The fitted average lifetime of the K_2_Sr_0.88_Al_0.1_P_2_O_7_:0.02Cr^3+^ phosphor (15.9 μs) is longer than that of the K_2_Sr_0.98_P_2_O_7:_0.02Cr^3+^ phosphor (12.4 μs), which also confirms the presence of more symmetrical [Sr/AlO_6_] octahedron as found by the decreased *D*_dis_ values. Moreover, the room-temperature PL decay curves of the K_2_Sr_0.9‑*y*_Al_0.1_P_2_O_7_:*y*Cr^3+^ phosphor, collected under 448 nm excitation and presented in Figure S9, reveal a gradual decrease in calculated lifetimes from 16.5 to 15.4 μs with increasing Cr^3+^ concentration, suggesting an elevated possibility of nonradiative transitions at higher Cr^3+^ concentrations.

### Temperature-Dependent PL Properties

The thermal stability of our K_2_Sr_0.98_P_2_O_7_:0.02Cr^3+^ and K_2_Sr_0.88_Al_0.1_P_2_O_7_:0.02Cr^3+^ phosphors was evaluated through the analysis of temperature-dependent PL emission spectra, collected in the temperature range of 298 to 523 K under 448 nm excitation, as presented in Figure S10 and Figure 3a, respectively. The peak position and fwhm of the emission spectra of the K_2_Sr_0.88_Al_0.1_P_2_O_7_:0.02Cr^3+^ phosphor as shown in Figure S11 exhibit red-shift and broadening, respectively. The red shift of the PL spectra is related to the weakening of crystal field around Cr^3+^ due to the thermal expansion of the lattice, while the PL spectral broadening should be ascribed to the increased vibrational levels at higher temperature. The integral PL intensity first increases and then gradually decreases with increasing temperature (Figure 3b). The increase in total intensity exceeding unity, as observed in Figure 3b, is likely attributable to a thermally induced enhancement in the absorption probability of the ^4^A_2_ → ^4^T_1_ transition in the K_2_Sr_0.88_Al_0.1_P_2_O_7_:0.02Cr^3+^ phosphor (Figure S12) compared with the K_2_Sr_0.98_P_2_O_7_:0.02Cr^3+^ counterpart (Figure S13).^37^ When heated to 423 K, the integral emission intensity of K_2_Sr_0.88_Al_0.1_P_2_O_7_:0.02Cr^3+^ is 120% of that at room temperature while the integral emission intensity of K_2_Sr_0.98_P_2_O_7_:0.02Cr^3+^ remains 63% of that at room temperature. This value is high among Cr^3+^-activated phosphors (Table 1) and comparable with that of fluorides, such as ScF_3_:Cr^3+^ (*I*_423K_ = 85.5% *I*_300K_), suggesting that the K_2_Sr_0.88_Al_0.1_P_2_O_7_:0.02Cr^3+^ phosphor has outstanding antithermal quenching property.^38^ The thermal stability of the K_2_Sr_1–*x*_Al_*x*_P_2_O_7_:0.01Cr^3+^ (0.05 ≤ *x* ≤ 0.2) phosphors with various Al^3+^ substitution concentrations was further investigated, as shown in Figure S14. It is noticed that the thermal quenching behavior significantly reduces with Al^3+^ substitution, indicating that the incorporation of Al^3+^ enhances the K_2_SrP_2_O_7_:0.01Cr^3+^ phosphor’s resistance to thermal quenching.

**Table 1:** PL Properties of Cr^3+^-Activated Broadband NIR Phosphors

**Host**	***λ***_**ex**_ **(nm)**	***λ***_**em**_ **(nm)**	**fwhm (nm)**	***I*** **@T (K)**	**Δ** ***E*** **(eV)**	**ref**
CaLu_2_Al_4_SiO_12_	425	760	∼130	118%@423	/	^40^
MgGa_2_O_4_	420	708	∼50	117.5%@423	/	^41^
BaAl_4_Sb_2_O_12_	428	750	/	118%@423	/	^42^
Mg_4_Ta_2_O_9_	450	842	167	∼55%@373	/	^43^
Ca_4_HfGe_3_O_12_	460	840	150	60.1%@373	0.28	^44^
YGa_3_(BO_3_)_4_	430	770	128	80%@423	0.31	^45^
Gd_3_Ga_5_O_12_	448	731	95	92.7%@423	0.33	^46^
Mg_2_GeO_4_	470	940	∼180	50%@310	/	^47^
Sr_9_Ga(PO_4_)_7_	485	850	∼140	∼5%@423	/	^48^
KGaP_2_O_7_	460	815	127	56%@423	0.30	^49^
KAlP_2_O_7_	450	790	120	77%@423	0.29	^50^
NaInP_2_O_7_	480	856	133	∼20%@423	0.33	^51^
NaGaP_2_O_7_	460	793	115	85.5%@423	0.30	^52^
LiInP_2_O_7_	460	860	165	22%@423	/	^53^
K_2_Sr_0.88_Al_0.1_P_2_O_7_	448	807	130	120%@423	0.92	This work

**Figure 3:**
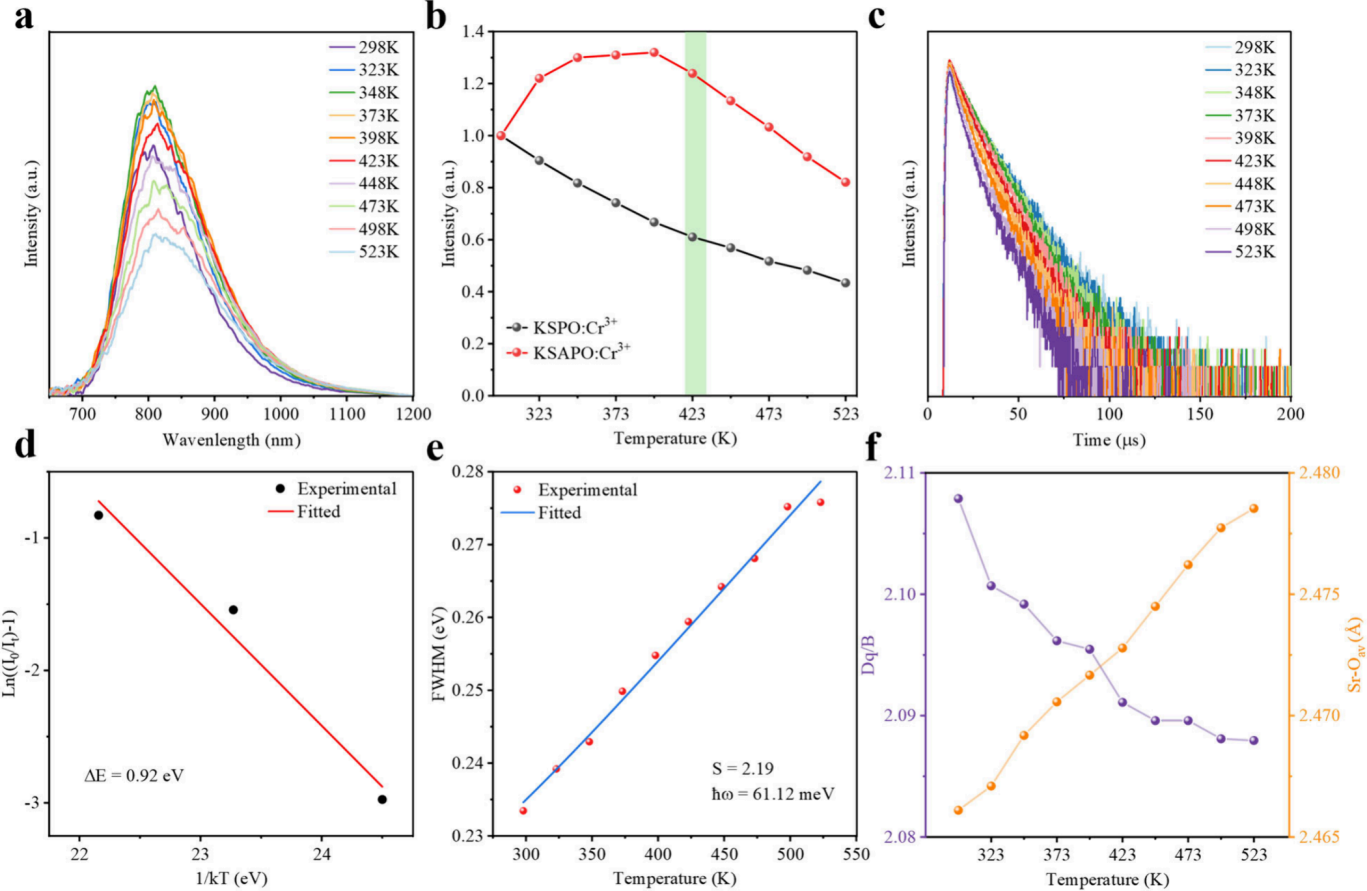
(a) Temperature-dependent PL spectra of the K_2_Sr_0.88_Al_0.1_P_2_O_7_:0.02Cr^3+^ phosphor measured in the temperature range of 298 – 523 K and in the wavelength range of 650 – 1200 nm (λ_ex_ = 448 nm). (b) Temperature-dependent integrated emission intensity of the K_2_Sr_0.98_P_2_O_7_:0.02Cr^3+^ and K_2_Sr_0.88_Al_0.1_P_2_O_7_:0.02Cr^3+^ phosphors labeled as KSPO:Cr^3+^ and KSAPO:Cr^3+^, respectively. (c) Lifetime decays, (d) plot of the relationship between *ln*(*I*_0_/*I*_*T*_ −1) versus 1/*kT* in the temperature range of 473 – 523 K, (e) plot of the temperature dependence of the fwhm, and (f) the *D*_*q*_/*B* value and the average Sr–O bond length as a function of temperature of the K_2_Sr_0.88_Al_0.1_P_2_O_7_:0.02Cr^3+^ phosphor measured in the temperature range of 298 – 523 K.

Heterovalent substitution strategies, such as the replacement of divalent cations with trivalent cations, have been employed to enhance the thermal stability of the phosphors. These substitutions stabilize the host lattice, by minimizing lattice vibrations-induced energy loss and forming more rigid crystal structures, thereby mitigating thermal quenching, as reported for Zn_1–*x*_Ga_2+*x*_O_4+δ_:Cr^3+^, Sr_1–*x*_La_1+*x*_ZnO_3.5+*x*/2_:Bi^3+^ (0 ≤ *x* ≤ 0.4), and Mg_1‑*x‑y*_Ga_*y*_O:*x*Cr^3+^.^18,32,39^ While previous studies have demonstrated that such approaches significantly reduce thermal quenching, however, the strategy utilized in this work has led to an antithermal quenching effect, as listed in Table S4. Further investigation of these findings in the context of the existing literature could provide deeper insights into the underlying mechanisms contributing to the observed thermal stability improvements in this study.

Generally, antithermal quenching of phosphors is closely related to the release of electrons from defect-induced trap states at increased temperatures. However, the thermoluminescence (TL) spectrum of the K_2_Sr_0.88_Al_0.1_P_2_O_7_:0.02Cr^3+^ phosphor (Figure S15) does not support the existence of defects. To reveal the mechanisms of such an outstanding thermal stability of this phosphor, we evaluated its structure rigidity through specific heat (*C*_p_) measurements of the host materials without Cr^3+^ dopant (Figure S16).^54^ The Debye temperature (Θ_D_), which commonly represents the structural rigidity of phosphors, was derived from the fitting of the specific heat data. The Θ_D_ values of the K_2_SrP_2_O_7_ and K_2_Sr_0.9_Al_0.1_P_2_O_7_ compounds were 335 and 318 K, respectively. While these Θ_D_ values are relatively low compared to compounds with a dense structural framework (Θ_D_ > 700 K) that exhibit higher rigidity, they still indicate adequate structural robustness to maintain thermal stability. Furthermore, previous research has shown that the Debye temperature is a useful parameter but not always an ideal representative for predicting the thermal quenching of phosphors.^55,56^

The temperature-dependent decay curves were also measured to study the thermal quenching behavior of the emission centers of the K_2_Sr_0.98‑*x*_Al_*x*_P_2_O_7_:0.02Cr^3+^ (*x* = 0 and 0.1) phosphors. Figure 3c and Figure S17 show the average lifetimes as a function of temperature of the K_2_Sr_0.88_Al_0.1_P_2_O_7_:0.02Cr^3+^ and K_2_Sr_0.98_P_2_O_7_:0.02Cr^3+^ phosphors, respectively. The average lifetime exhibits a slowly decreasing trend owing to the thermally induced nonradiative transition. In addition, as shown in Table S5, the decay rates of the K_2_Sr_0.88_Al_0.1_P_2_O_7_:0.02Cr^3+^ phosphor are lower than the K_2_Sr_0.98_P_2_O_7_:0.02Cr^3+^ phosphor, indicating that with Al substitution, the thermal-induced nonradiative transition was suppressed, which agrees with the fact that the K_2_Sr_0.88_Al_0.1_P_2_O_7_:0.02Cr^3+^ phosphor showed higher thermal resistance.

The thermal quenching behavior is illustrated by the configuration coordinate diagram in Figure S18. With x = 0 taken as an example, the O→A and B→C transitions correspond to standard excitation and emission processes, respectively. As the temperature rises, excited electrons gain thermal energy, enabling them to reach the intersection of the ^4^T and ^4^A energy curves with corresponding B→C transition. Subsequently, they return to the ^4^A ground state via a nonradiative C→O transition, resulting in a thermal quenching phenomenon. The activation energy for thermal quenching (Δ*E*) represents the energy barrier associated with the thermally activated process. It quantifies the difficulty of thermal quenching by defining the energy difference between the lowest excited-state energy and the crossing point of the ground state. This parameter was calculated according to the Arrhenius equation:^57^

6 $$I=\frac{I_{0}}{1+A\times e^{-\Delta E/kT}}$$

where *I* and *I*_0_ represent the integral emission intensity at temperature *T* and initial emission intensity at room temperature, *A* is the constant for host lattice, and *k* is the Boltzmann’s constant. As shown in Figure 3d, the Δ*E* of the K_2_Sr_0.88_Al_0.1_P_2_O_7_:0.02Cr^3+^ phosphor is estimated to 0.92 eV, which is much higher than K_2_Sr_0.98_P_2_O_7_:0.02Cr^3+^ (0.18 eV, Figure S19). This activation energy is exceptionally high compared to other Cr^3+^-doped phosphors reported in the literature, as demonstrated in Table 1.To the best of our knowledge, this represents the highest Δ*E* reported to date for Cr^3+^-doped phosphors.^58^ These results demonstrate that there is a relatively high energy barrier to resist thermal quenching after partially substituting Sr^2+^ with Al^3+^ into the K_2_Sr_0.98_P_2_O_7_:0.02Cr^3+^ sample. However, we note that due to the requirement of *I* < *I*_0_ for valid Arrhenius fitting, only three data points at and above 473 K were used.

In addition, Huang’s theory points out that the activation energy is dependent on the strength of electron–phonon coupling, which can be reflected by the Huang–Rhys factor (*S*). The smaller Stokes shift of the K_2_Sr_0.88_Al_0.1_P_2_O_7_:0.02Cr^3+^ phosphor indicates weak EPC (Figure S18). The EPC effect of the K_2_Sr_0.98_P_2_O_7_:0.02Cr^3+^ and K_2_Sr_0.88_Al_0.1_P_2_O_7_:0.02Cr^3+^ phosphors can be obtained by fitting the following equation:^58^

7 $$FWHM\left(T\right)=\sqrt{8\times \ln2}\times \sqrt{S}\times \hbar \omega \times \sqrt{\coth\frac{\hbar \omega }{2\times k\times T}}$$

where the fwhm (*T*) is obtained from fitting each emission spectra at measurement temperatures *T*, *ℏω* is the phonon energy, and *k* is the Boltzmann’s constant (8.617 × 10^–5^ eV/K). As shown in Figures S20 and 3e, the *ℏω* values obtained for the K_2_Sr_0.98_P_2_O_7_:0.02Cr^3+^ and K_2_Sr_0.88_Al_0.1_P_2_O_7_:0.02Cr^3+^ phosphors are 44.66 and 61.12 meV, respectively. The *S* values of the K_2_Sr_0.98_P_2_O_7_:0.02Cr^3+^ and K_2_Sr_0.88_Al_0.1_P_2_O_7_:0.02Cr^3+^ phosphors are found to be 3.22 and 2.19, respectively. The smaller *S* value for the latter after Al^3+^ substitution implies that it has a larger activation energy and higher thermal quenching temperature by reducing the phonon-assisted nonradiative relaxation process at high temperatures.

Notably, the *S* value of the K_2_Sr_0.88_Al_0.1_P_2_O_7_:0.02Cr^3+^ phosphor is much smaller than that observed in previously reported Cr^3+^-activated materials, such as Ca_3_Sc_2_Si_3_O_12_:Cr^3+^ (*S* = 4.0), GaTaO_4_:Cr^3+^ (*S* = 3.8), and Cs_2_AgInCl_6_:Cr^3+^ (*S* = 3.2), indicating that the EPC effect of the K_2_Sr_0.88_Al_0.1_P_2_O_7_:0.02Cr^3+^ is extremely weak.^59–61^ The weak EPC means that the excited electrons can be hardly affected by phonons to reach the crossing point between excited and ground states.

To further explore the temperature-dependent stable emission peaks of the K_2_Sr_0.88_Al_0.1_P_2_O_7_:0.02Cr^3+^ phosphor, the crystal field splitting energy *D*_*q*_ at different temperatures was obtained by spectral calculation. It can be found that such a huge temperature difference has little effect on the crystal field (Figure 3f). Rietveld refinements of the temperature-dependent XRD analyses for both the K_2_Sr_0.88_Al_0.1_P_2_O_7_:0.02Cr^3+^ and K_2_Sr_0.98_P_2_O_7_:0.02Cr^3+^ phosphors are shown in Figures S21 and S22, respectively. The corresponding variation of lattice parameters with the temperature is presented in Figure S23. Throughout the temperature range of 298–523 K, both samples retain their original crystal phase. A gradual lattice expansion is observed for both samples along with a slight increase in the average Sr–O bond lengths, as shown in Figures 3f and S24. While the K_2_Sr_0.88_Al_0.1_P_2_O_7_:0.02Cr^3+^ phosphor shows a smooth bond-length increase, the K_2_Sr_0.98_P_2_O_7_:0.02Cr^3+^ sample exhibits a more abrupt and irregular trend, indicating distinct thermal adaptation behaviors. Moreover, the thermal expansion coefficients of the K_2_Sr_0.88_Al_0.1_P_2_O_7_:0.02Cr^3+^ and K_2_Sr_0.98_P_2_O_7_:0.02Cr^3+^ samples are shown in Figure S25. The K_2_Sr_0.88_Al_0.1_P_2_O_7_:0.02Cr^3+^ phosphor exhibits a higher overall expansion coefficient than the K_2_Sr_0.98_P_2_O_7_:0.02Cr^3+^ counterpart, consistent with its lower Debye temperature.^62^ Unlike the K_2_Sr_0.98_P_2_O_7_:0.02Cr^3+^ phosphor, which shows a continuous increase with temperature, the K_2_Sr_0.88_Al_0.1_P_2_O_7_:0.02Cr^3+^ phosphor displays a suppressed expansion rate between 348 and 448 K. Although a partial recovery is observed at higher temperatures, the expansion coefficient remains below that at 323 K. This behavior suggests that heterovalent substitution enhances the structure resilience of the K_2_Sr_0.88_Al_0.1_P_2_O_7_:0.02Cr^3+^ phosphor upon heating.

The suppression of thermal quenching can be attributed to the interplay of multiple contributing factors. The widened band gap increased the energy separation between the valence and conduction bands, requiring excited electrons to overcome a larger barrier for thermal ionization or charge transfer. This leads to a reduction in nonradiative recombination and an enhancement in thermal stability. Additionally, an increased thermally induced absorption probability improves energy utilization, while a higher energy barrier suppresses nonradiative transitions. Furthermore, the minimal EPC weakens energy loss through lattice vibrations, the limited effect of thermal excitation on the crystal field preserves the electronic environment, and the stability of the Sr–O bond length across temperature variations maintains structural integrity. Together, these mechanisms synergistically inhibit thermal quenching, providing insight into the K_2_Sr_0.88_Al_0.1_P_2_O_7_:0.02Cr^3+^ phosphor’s robust luminescence performance under thermal stress, consistent with prior reports of Cr^3+^-based systems.^14,63^ Although Debye temperature analysis suggests a slight decrease in lattice rigidity upon substitution (from 335 to 318 K), this is counterbalanced by a lower Huang–Rhys factor (*S* = 2.19), signifying weaker EPC and a reduced probability of phonon-assisted nonradiadiative transitions. The combination of moderate lattice rigidity and weak EPC effectively suppresses thermal deactivation in the K_2_Sr_0.88_Al_0.1_P_2_O_7_:0.02Cr^3+^ phosphor. Thus, the heterovalent substitution effectively induces band gap widening, suppressing nonradiative relaxation and thermal ionization, and ensures sufficient structural robustness, thereby endowing the K_2_Sr_0.88_Al_0.1_P_2_O_7_:0.02Cr^3+^ phosphor with exceptional thermal stability.

### Applications for NIR pc-LEDs

Owing to the remarkable spectral variability and superior PL characteristics of the K_2_Sr_0.88_Al_0.1_P_2_O_7_:0.02Cr^3+^ phosphor, NIR pc-LEDs were effectively constructed by depositing them onto commercially available 450 nm blue LED chips. The electroluminescence spectra of the NIR pc-LED incorporating the K_2_Sr_0.88_Al_0.1_P_2_O_7_:0.02Cr^3+^ phosphor are presented in Figure S26, revealing visible emission from the LED chip at ∼ 450 nm and a broadband emission spanning 650–1100 nm. When the driving current increases from 50 to 350 mA, the intensity of NIR emission bands increases, showing no sign of saturation due to the excellent luminescence thermal stability of the NIR K_2_Sr_0.88_Al_0.1_P_2_O_7_:0.02Cr^3+^ phosphor. In comparison, many previously optimized phosphors via heterovalent substitution strategies have been constrained by thermal stability limitations, thereby restricting their applicability in high-power LED chips under elevated operational currents.^18,27,32,64^ The ability of our materials to sustain efficient and stable NIR emission under higher driving currents makes it a potential candidate for high-power lighting applications.

Driven by the exceptional luminescence potential of the NIR pc-LED, a variety of innovative applications have been envisioned, as illustrated in Figure 4. In conjunction with an NIR camera, the NIR pc-LED is capable of swiftly and accurately revealing hidden information. Illustratively, Figure 4a exemplifies two instances of the numeral sequence “8888” written on white paper: the first sequence contains hidden information penciled in, whereas the second sequence was obscured with a marker. When illuminated by the fluorescent lamp, there is little visual distinction between the two sets of numerals to the naked eye. Conversely, when illuminated by the NIR pc-LED light, the true messages “1192” and “IECH,” are clearly disclosed, as captured by the NIR camera. This enhanced visibility is ascribed to the differing absorption properties of the NIR light by pencil (strong absorption) and black watercolor (weak absorption), as shown in Figures 4c and 4b, respectively. Further demonstrating the capabilities of this technology, Figure 4b includes an image of a card covered with sunglasses captured under the fluorescent lamp light using a digital camera. The NIR emission from the LED penetrates the sunglass with ease, allowing clear visualization of the text “KSAPO:Cr” through an NIR camera, as depicted in Figure 4d. This example highlights the potential of the NIR pc-LED for noninvasive investigative applications.

**Figure 4:**
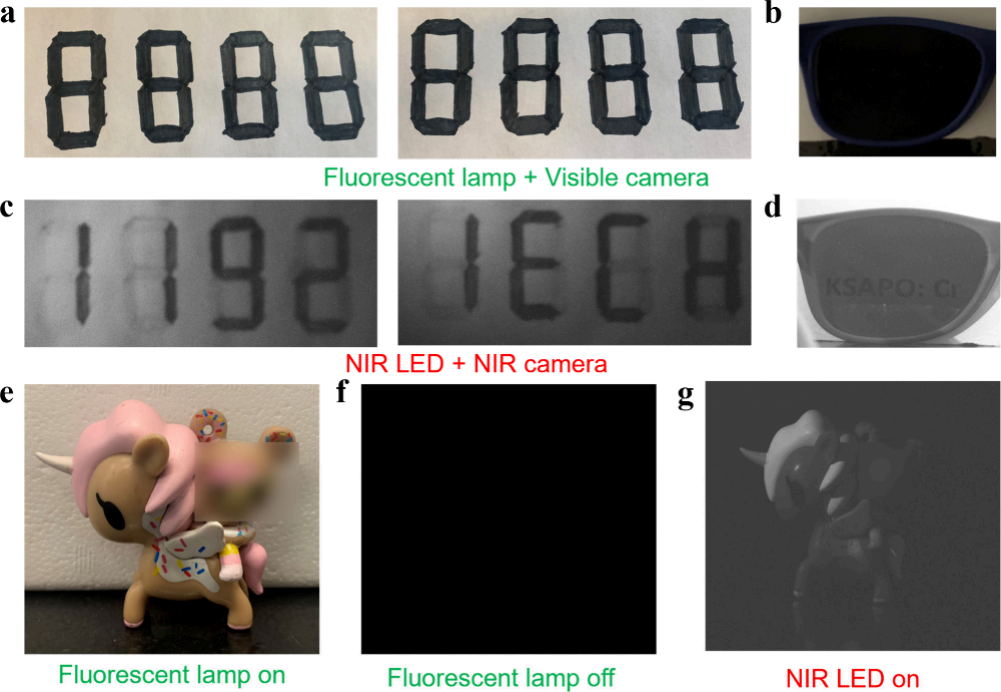
(a and b) Visible images with a visible camera with a fluorescent lamp. (c and d) Images taken using a NIR camera with NIR pc-LED was on. (e-g) Images of a toy illuminated with fluorescent lamp and NIR LED.

Moreover, the application of NIR pc-LED in night vision technologies has been investigated, as detailed in Figures 4e and 4g. When illuminated with a standard fluorescent lamp, the image of a toy displayed its original colors and was clearly visible (Figure 4e). However, this image became indistinct once the fluorescent light source was turned off (Figure 4f). An NIR camera equipped with a 550 nm long-pass filter coupled with the NIR pc-LED as an illumination source enabled clear imaging of the toy in the absence of visible light by effectively blocking residual blue light from the LED chip (Figure 4f). These results underscore the remarkable potential of the K_2_Sr_0.88_Al_0.1_P_2_O_7_:0.02Cr^3+^ phosphors as effective NIR light-emitting sources, indicating substantial progress in the field of high-performance NIR illumination applications.

## Conclusions

In summary, Cr^3+^-doped K_2_Sr_1–*x*_Al_*x*_P_2_O_7_ phosphors were synthesized, achieving simultaneous improvements in the emission intensity and thermal stability through Sr/Al substitution. The optimized phosphor, K_2_Sr_0.88_Al_0.1_P_2_O_7_:0.02Cr^3+^, efficiently emits broadband NIR light ranging from 650 to 1100 nm when excited by 448 nm. At 423 K, the emission intensity retains 120% of its initial value at room temperature, demonstrating exceptional thermal stability with antithermal-quenching property. Structural and spectral analyses reveal that the remarkable thermal stability of Cr^3+^ luminescence emission in the K_2_Sr_0.9_Al_0.1_P_2_O_7_ host is significantly enhanced by Sr^2+^ ↔ Al^3+^ heterovalent substitution, driven by a widened band gap, ultraweak EPC, increased thermally induced absorption probability, a fortified energy barrier, and an optimized local symmetry within the [Sr/AlO_6_] octahedron sites. Temperature-dependent XRD analysis confirms that Al^3+^ substitution reduces the extent of lattice expansion upon heating, thereby enhancing the structural stability against thermal stress. Furthermore, an NIR pc-LED incorporating this NIR phosphor was developed, showing promising potential for information recognition, nondestructive analysis, night vision, and related applications. This study provides an example of a heterovalent substitution strategy for enhancing the performance of Cr^3+^-based near-infrared phosphors in advanced NIR photonic applications.

## Supplementary Material



## Notes

XRD patterns; detailed Rietveld refinement results; SEM and EDX analyses; absorption and diffuse reflectance spectra; additional PL and PLE data; quantum efficiency; decay curves; plot of ln­[(*I*_0_/*I*_T_) – 1] versus 1/*kT*; temperature-dependent PL and PLE emission spectra; thermoluminescence spectra; heat capacity measurements; and electroluminescence spectra (PDF)
