# Supplementary material for: Heterovalent Substitution of K2SrP2O7:Cr3+ to Achieve Anti-Thermal-Quenching Broadband Near-Infrared Luminescence
Source: ACS Appl Opt Mater. 2025 Aug 11;3(8):1766–76. doi: 10.1021/acsaom.5c00201 (PMC12379184; doi:10.1021/acsaom.5c00201)
Supplement: Supplementary file 1 [file ot5c00201_si_001.pdf]

Supporting Information

Heterovalent Substitution of $K_2SrP_2O_7:Cr^{3+}$ to Achieve Anti-Thermal-Quenching Broadband Near-Infrared Luminescence

Hexi Zhang and Yuanbing Mao*

Department of Chemistry, Illinois Institute of Technology, Chicago, IL 60616, USA

*To whom correspondence should be addressed: ymao17@iit.edu, Tel.: +1-312-567-3815

Table S1. Refined structural data of $K_2Sr_{0.88}Al_{0.10}P_2O_7:0.02Cr^{3+}$ sample

Crystal system	Monoclinic
Space group	$P2_1/c$
a (Å)	9.17198
b (Å)	5.71952
c (Å)	14.72223
V (Å^3)	743.452
$\alpha = \gamma$ (°)	90
β (°)	105.714
R_p (%)	5.66
R_{wp} (%)	7.14
χ^2	2.53

Table S2. Sr-O bond lengths (Å) of $K_2Sr_{0.99-x}Al_xP_2O_7$:$0.01Cr^{3+}$ ($0.05 \leq x \leq 0.2$) phosphors

$x = 0$	
Sr-O1	2.333
Sr-O2	2.695
Sr-O3	2.474
Sr-O4	2.534
Sr-O5	2.394
Sr-O6	2.394
$x = 0.005$	
Sr-O1	2.424
Sr-O2	2.586
Sr-O3	2.404
Sr-O4	2.393
Sr-O5	2.415
Sr-O6	2.554
$x = 0.10$	
Sr-O1	2.434
Sr-O2	2.585
Sr-O3	2.434
Sr-O4	2.393
Sr-O5	2.505
Sr-O6	2.513
$x = 0.15$	
Sr-O1	2.424
Sr-O2	2.505
Sr-O3	2.434
Sr-O4	2.393
Sr-O5	2.505
Sr-O6	2.494
$x = 0.2$	
Sr-O1	2.324
Sr-O2	2.4195
Sr-O3	2.405
Sr-O4	2.423
Sr-O5	2.505
Sr-O6	2.404

Table S3. Crystal field intensity of the $K_2Sr_{0.99-x}Al_xP_2O_7$:$0.01Cr^{3+}$ ($0.05 \le x \le 0.2$) phosphors

x	0	0.005	0.1	0.15	0.2
Dq/B	2.1	2.15	2.14	2.13	2.13

Table S4. Photoluminescence properties of optimized phosphors with heterovalent substitution

Phosphor	λ_{ex} (nm)	λ_{em} (nm)	IQE	I@T(K)	Ref
$Zn_{0.98}Ga_{0.02}O_{4+\delta}$:$Cr^{3+}$	400	690	/	77.3%@423K	1
$Sr_{0.6}La_{1.4}ZnO_{3.7}$:$Bi^{3+}$	390	609	60%	13%@423K	2
$Mg_{0.985}Ga_{0.0005}O$:Cr^{3+}	468	862	30%	65%@423K	3
$K_{1.933}Hf_{0.867}Lu_{0.2}Si_3O_9$:$Eu^{2+}$	365	496	/	93.8%@473K	4
$Ba_2Si_{0.97}Al_{0.03}O_{3.985}$:$Eu^{2+}$	450	508	53.81%	88.7%@373K	5
$CaSi_{1.96}Al_{0.04}O_7$:Eu^{3+}	394	617	/	79.36%@483K	6
$Cs_3Zn_{0.95}Al_{0.05}Cl_5$:$Cu^{2+}$	266	484	93.60%	/	7
$Ca_9LiMg_{1/2}Al_{1/3}(PO_4)_7$:$Eu^{2+}$	350	415	13.80%	52.7%@425K	8
$K_2Sr_{0.9}Al_{0.1}P_2O_7$:$Cr^{3+}$	448	807	40.10%	120%@423K	This work

Table S5. Decay rate of $K_2Sr_{0.98}P_2O_7$:$0.02Cr^{3+}$ and $K_2Sr_{0.88}Al_{0.1}P_2O_7$:$0.02Cr^{3+}$ phosphors with different temperatures

Temperature (K)	Decay rate (1/μs)	
	$K_2Sr_{0.88}Al_{0.1}P_2O_7$:$0.02Cr^{3+}$	$K_2Sr_{0.98}P_2O_7$:$0.02Cr^{3+}$
298	0.06	0.08
323	0.07	0.10
348	0.08	0.13
373	0.08	0.14
398	0.09	0.15
423	0.10	0.17
448	0.10	0.17
473	0.11	0.17
498	0.12	0.16
523	0.12	0.23

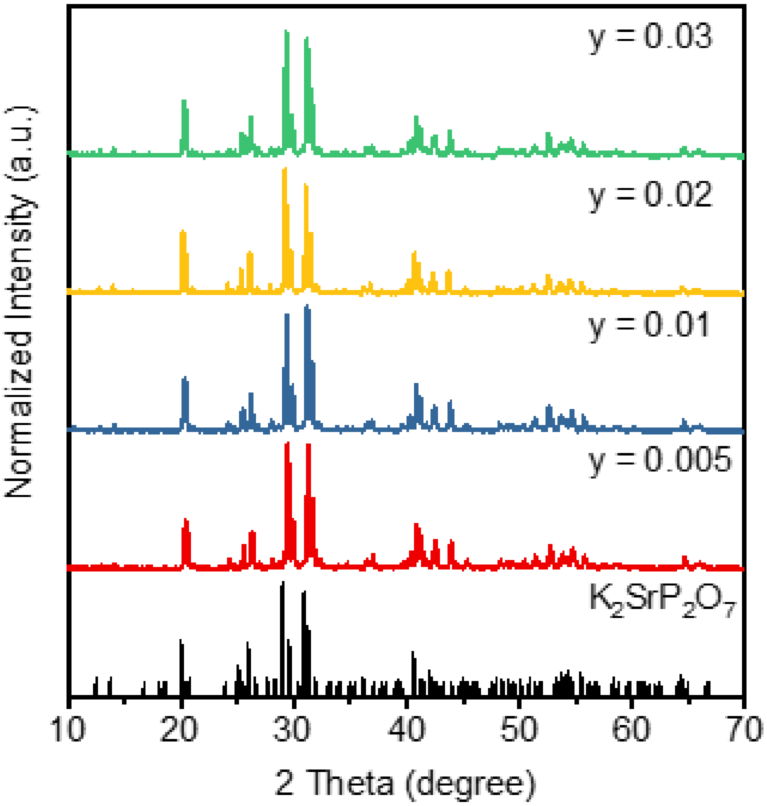

Figure S1. XRD patterns of the $K_2Sr_{0.9\text{-}y}Al_{0.1}P_2O_7$:$y$$Cr^{3+}$ (y = 0.005, 0.01, 0.02 and 0.03) phosphors. The standard XRD card of K_2SrO_7 (PDF card No. 01-077-0727) is used as reference.

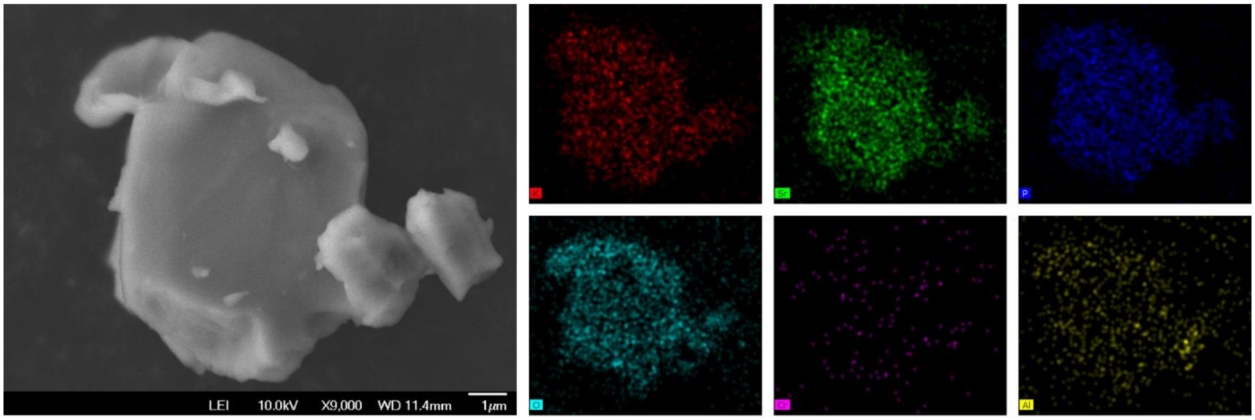

Figure S2. SEM image and EDS mapping images of the $K_2Sr_{0.88}Al_{0.1}P_2O_7$:0.02$Cr^{3+}$ phosphor.

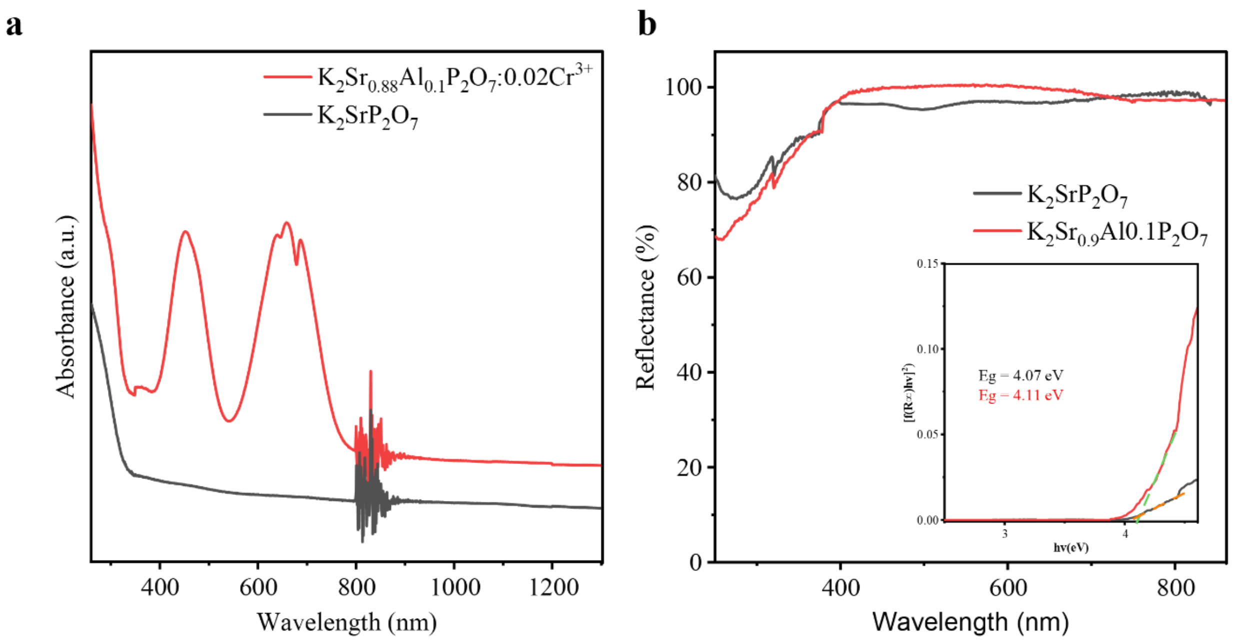

Figure S3. (a) Absorption spectra of the $K_2Sr_{0.88}Al_{0.1}P_2O_7:0.02Cr^{3+}$ and $K_2SrP_2O_7$ samples. Peaks around 800 nm region are spectroscopic instrument noise. (b) Diffuse reflectance spectra of the $K_2SrP_2O_7$ and $K_2Sr_{0.9}Al_{0.1}P_2O_7$ samples. The insect shows their corresponding optical band gaps.

The Kubelka-Munk function,

$$f(R_\infty) = \frac{K}{S} = \frac{absorption\ coefficient}{scattering\ coefficient} \quad (S1)$$

The optical band gap can be obtained by the following equation by Tauc.

$$[f(R_\infty)hv]^{1-n} = A(hv - E_g) \quad (S2)$$

Where A is a constant, hv is the photon energy, E_g (eV) is the optical band gap and n is the coefficient associated with the electronic transition type. By plotting $[f(R_\infty)\ hv]^{1/n}$ as a function of hv, E_g is obtained from the intersection of the extrapolated tangent line of the plot along the hv axis.

The apparent reflectance values slightly exceeding 100% are due to instrumental baseline noise and do not represent physically meaningful data. The trend remains reliable for optical band gap analysis.

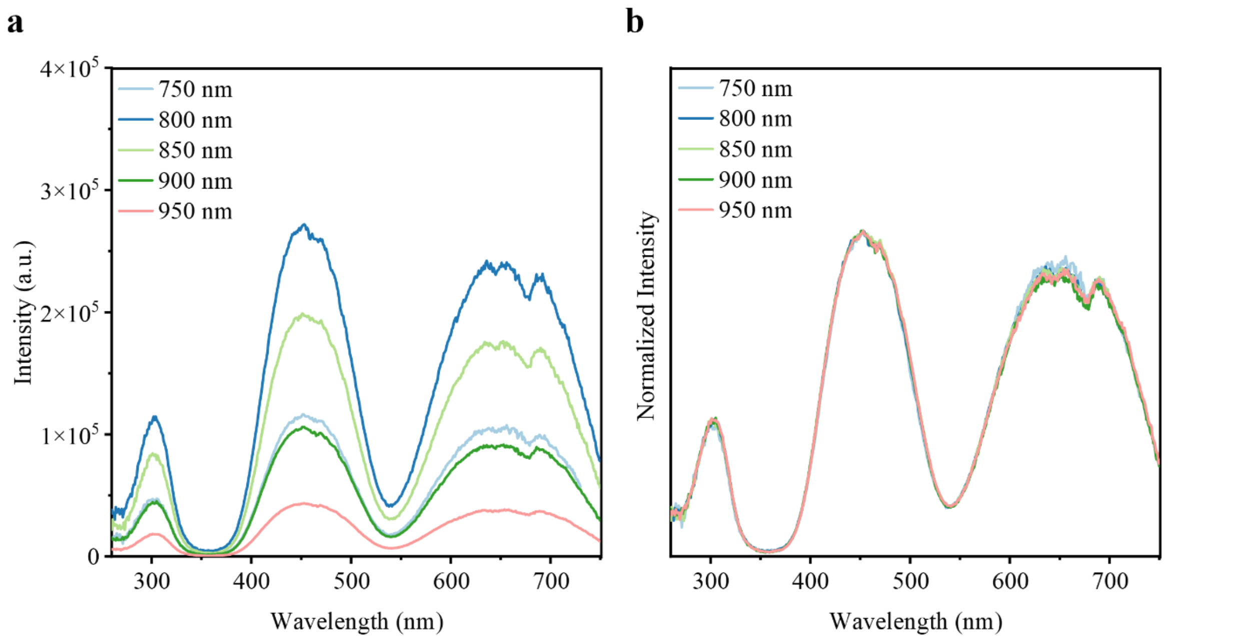

Figure S4. (a) Measured and (b) normalized excitation spectra of the $K_2Sr_{0.88}Al_{0.1}P_2O_7$:0.02$Cr^{3+}$ phosphor at different emission spectral positions.

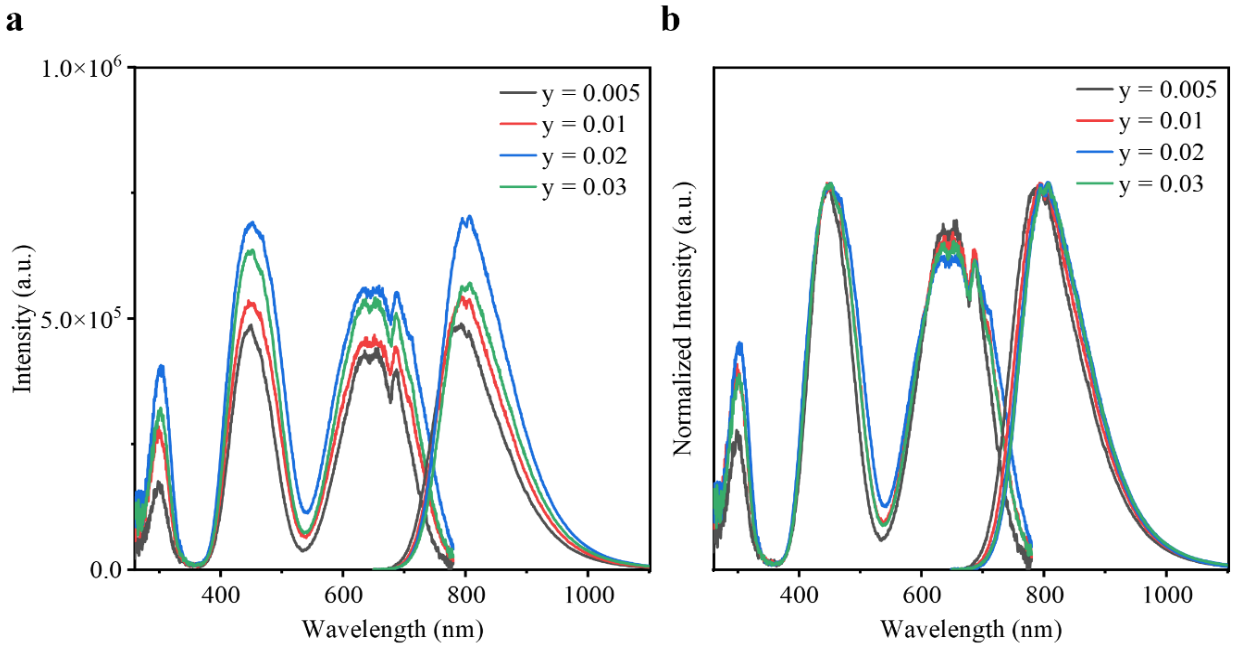

Figure S5. (a) Measured and (b) normalized PLE and PL spectra of the $K_2Sr_{0.9\text{-}y}Al_{0.1}P_2O_7$:$y$$Cr^{3+}$ (y = 0.005, 0.01, 0.02 and 0.03) phosphors.

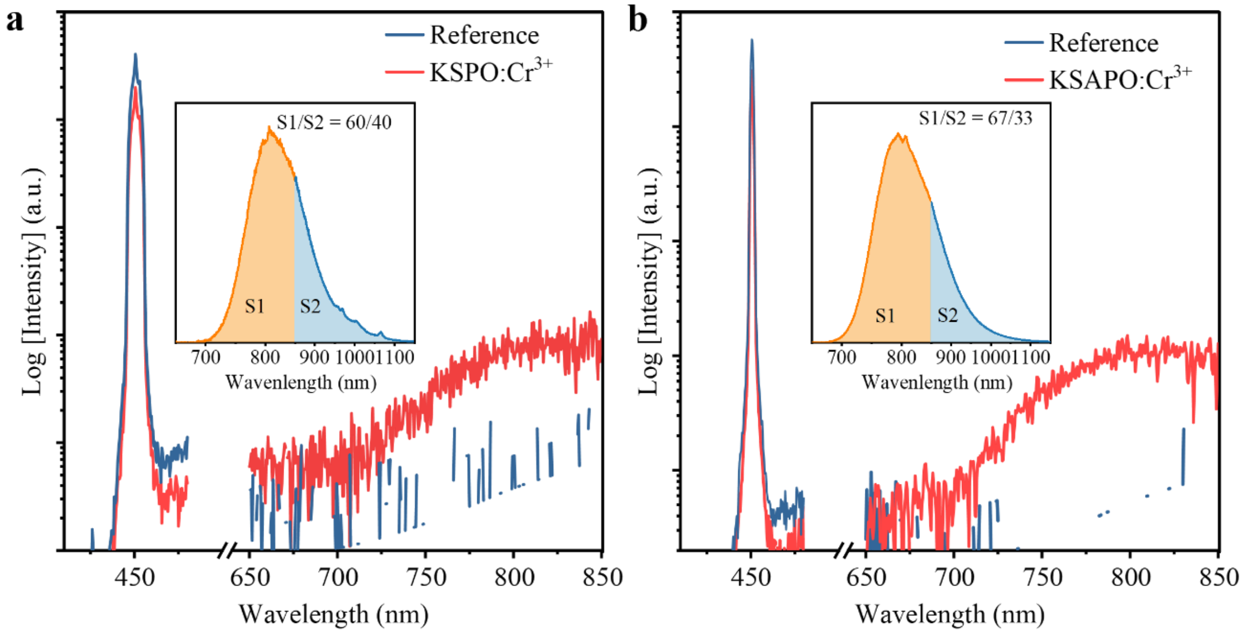

Figure S6. Quantum efficiency measurement of the (a) $K_2Sr_{0.98}P_2O_7$:0.02Cr^{3+} (labeled as KSPO:Cr^{3+}) and (b) $K_2Sr_{0.88}Al_{0.1}P_2O_7$:0.02$Cr^{3+}$ (labeled as KSAPO:Cr^{3+}) phosphors under 448 nm excitation.

The internal quantum efficiency (IQE) evaluation for the KSAPO:0.02Cr^{3+} and KSPO:0.02Cr^{3+} phosphors were constrained to a measurement wavelength range of up to 850 nm. This limitation was imposed by the quantum efficiency (QE) measuring instrument utilized in the study. To accurately determine the actual IQE, an adjustment was made to the calculation to account for the emission range beyond 850 nm (designated as S2). In specific terms, the integrated emission spectrum of the KSAPO:0.02Cr^{3+} phosphor covers the range from 650 to 850 nm (S1), which accounted for 67% of the total emission, leaving the unmeasured range (S2) to represent 33% of the emission. Similarly, for the KSPO:0.02Cr^{3+} phosphor, the integrated emission spectra from 650 to 850 nm (S1) comprised 60% of the total emission, with the unmeasured range (S2) contributing to the remaining 40% of the total emission.

The absorption efficiency (ξ_{abs}), IQE and external quantum efficiency (EQE) were calculated used the following equations:

$$\xi_{abs} = \frac{\int E_R - \int E_S}{\int E_S} \quad \text{(S3)}$$

$$IQE = \frac{\int L_s}{\int E_R - \int E_s} \quad \text{(S4)}$$

$$EQE = IQE \times \xi_{abs} \quad \text{(S5)}$$

Here, $\int L_s$ denotes the integrated emission spectrum, while $\int E_R$ *and* $\int E_s$ correspond to the excitation spectra acquired without and with the phosphor in the integrating sphere, respectively. Based on the calculation, the ξ_{abs} and EQE for KSPO:0.02Cr^{3+} were determined to be 70.6% and 10.3%, respectively. For KSAPO:0.02Cr^{3+}, the corresponding values were 87.7% and 35.2%, demonstrating notable improvement following Al^{3+} substitution.

To evaluate the uncertainty in EQE, a 5% relative uncertainty was applied to the IQE values based on typical error ranges reported for integrating-sphere-based QE measurements.[9] The standard error ξ_{abs} was calculated from three independent integrations of the excitation spectra. The standard errors ξ_{abs} were estimated to be ~0.00013 for the KSAPO:0.02Cr^{3+} sample, and ~0.0000037 for the KSPO:0.02Cr^{3+} sample, respectively. Using standard error propagation, the final EQE uncertainties were determined to be ±1.8% for the KSAPO:0.02Cr^{3+} and ±0.5% for KSPO:0.02Cr^{3+} samples.

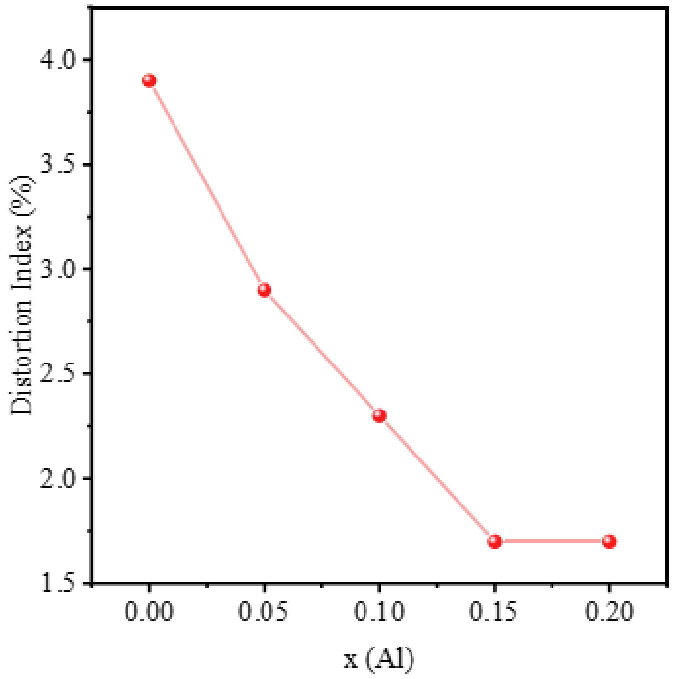

Figure S7. Distortion index of the [Sr/AlO6] octahedron of $K_2Sr_{0.99-x}Al_xP_2O_7$:0.01Cr^{3+} ($0.05 \le x \le 0.2$) phosphors.

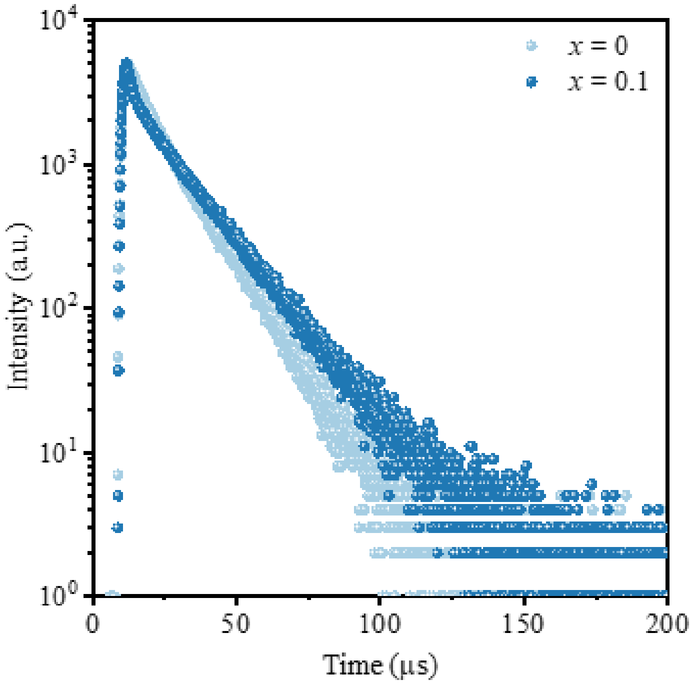

Figure S8. Room-temperature PL decay curves of the $K_2Sr_{0.98-x}Al_xP_2O_7$:0.02Cr^{3+} (x = 0 and 0.1) phosphors.

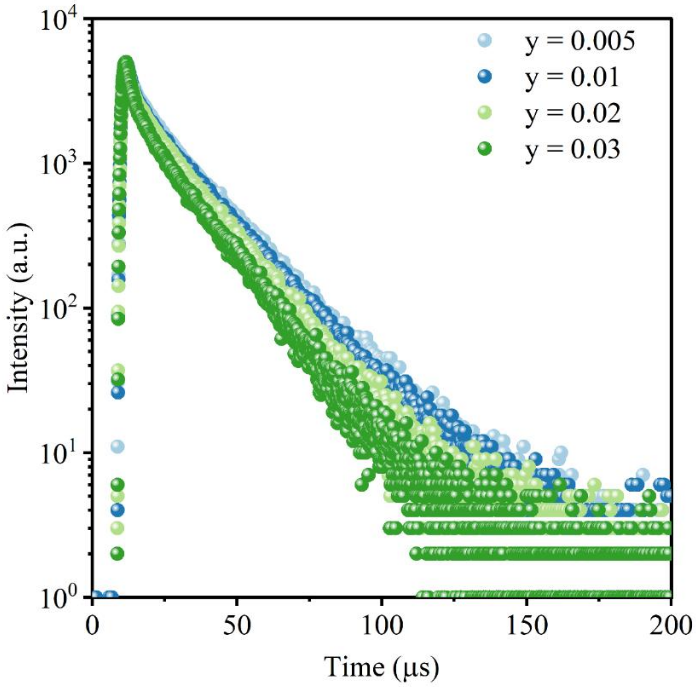

Figure S9. Room-temperature PL decay curves of the $K_2Sr_{0.9-y}Al_{0.1}P_2O_7$:$y$$Cr^{3+}$ (y = 0.005, 0.01, 0.02 and 0.03) phosphors.

The lifetime of Cr^{3+} are obtained by well fitted by a biexponential function are present:[10]

$$I = A_1 e^{-(t/\tau_1)} + A_2 e^{-(t/\tau_2)} \tag{S6}$$

$$\tau_{ave} = \frac{A_1\tau_1^2 + A_2\tau_2^2}{A_1\tau_1 + A_2\tau_2} \tag{S7}$$

where I represents the emission intensity, A_1 and A_2 are constants, τ_1 and τ_2 are the decay time for the exponential components, and τ_{ave} is the average decay time. The longer τ_2 regraded as the intrinsic Cr^{3+} ionic luminescence lifetime and the shorter τ_1 may be induced by additional energy decay paths like cross-relaxation or energy migration.[11, 12] The calculated decay time of the $K_2Sr_{0.9-y}Al_{0.1}P_2O_7{:}yCr^{3+}$ (y = 0.005, 0.01, 0.02 and 0.03) phosphors are 18.6 μs, 18.0 μs, 15.9 μs and 15.4 μs, respectively.

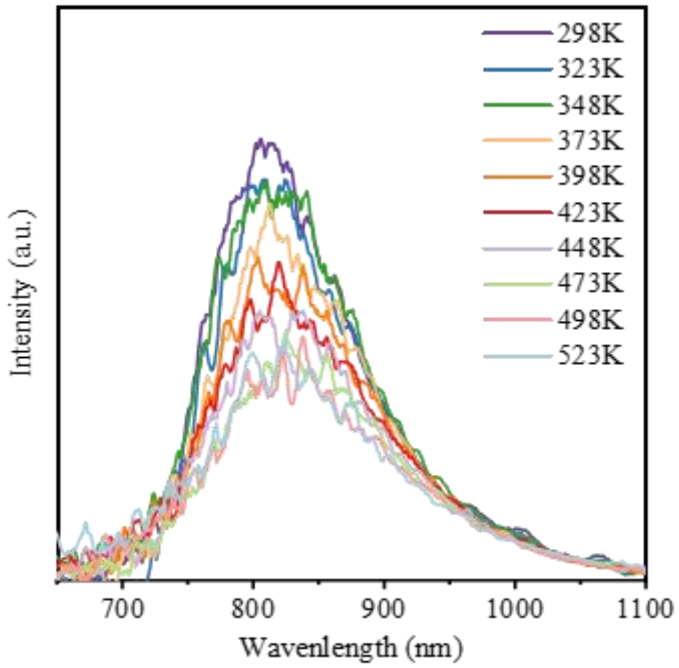

Figure S10. Temperature-dependent PL spectra of the $K_2Sr_{0.98}P_2O_7{:}0.02Cr^{3+}$ phosphor measured in the temperature range of 298-523K (λ_{ex} = 448 nm).

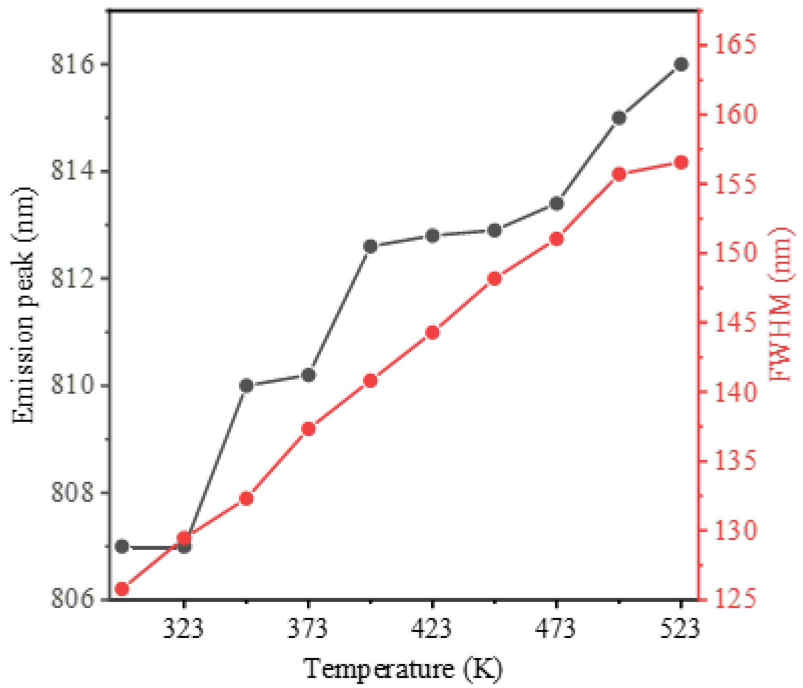

Figure S11. Temperature-dependent peak position and FWHM data for the $K_2Sr_{0.88}Al_{0.1}P_2O_7$:$0.02Cr^{3+}$ phosphor.

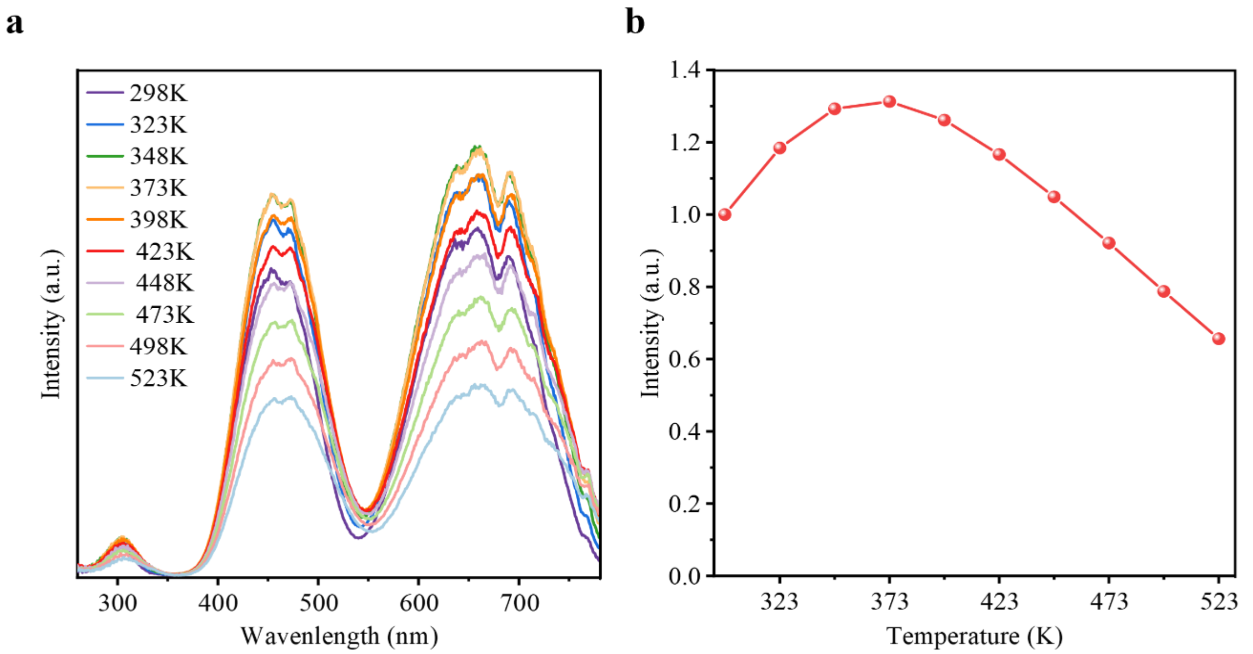

Figure S12. The $K_2Sr_{0.88}Al_{0.1}P_2O_7$:$0.02Cr^{3+}$ phosphor: (a) Temperature-dependent PLE spectra (λ_{em} = 807 nm). (b) Integrated PL excitation intensity *vs.* measurement temperature corresponding to the spectra in (a).

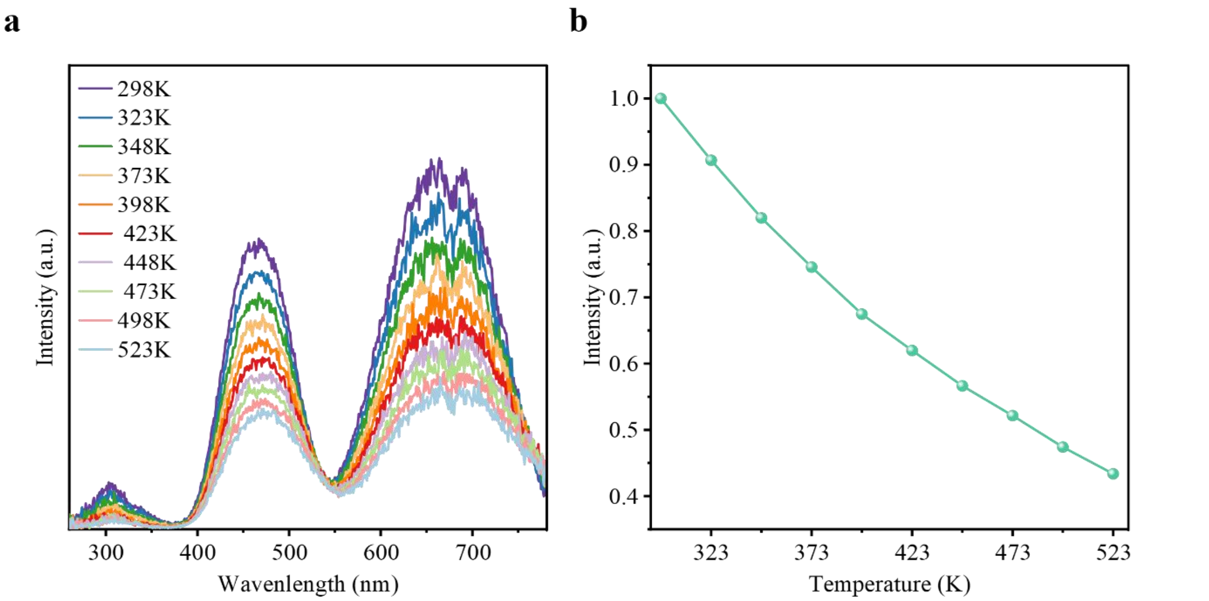

Figure S13. The $K_2Sr_{0.98}P_2O_7$:$0.02Cr^{3+}$ phosphor: (a) Temperature-dependent PLE spectra (λ_{em} = 813 nm). (b) Integrated PL excitation intensity *vs.* measurement temperature corresponding to the spectra in (a).

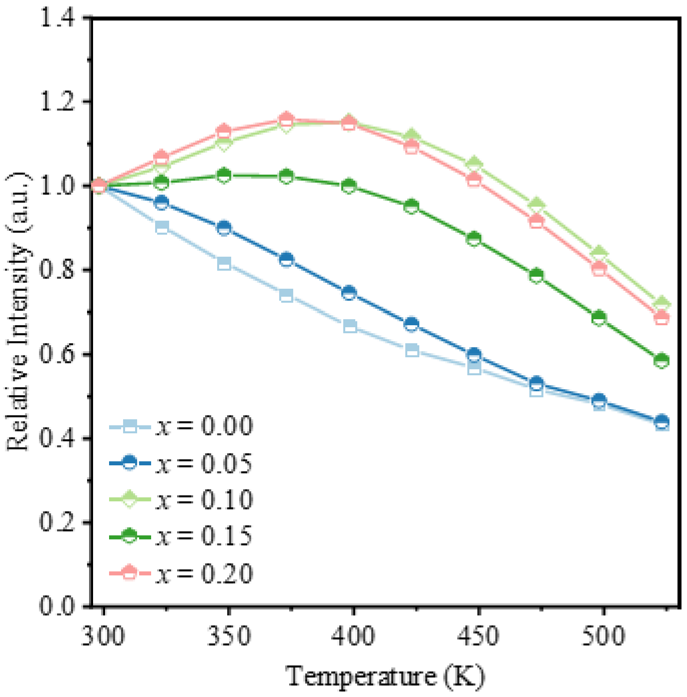

Figure S14. Temperature-dependent integrated emission intensity of the $K_2Sr_{0.99-x}Al_xP_2O_7$:$0.01Cr^{3+}$ ($0.05 \leq x \leq 0.2$) phosphors.

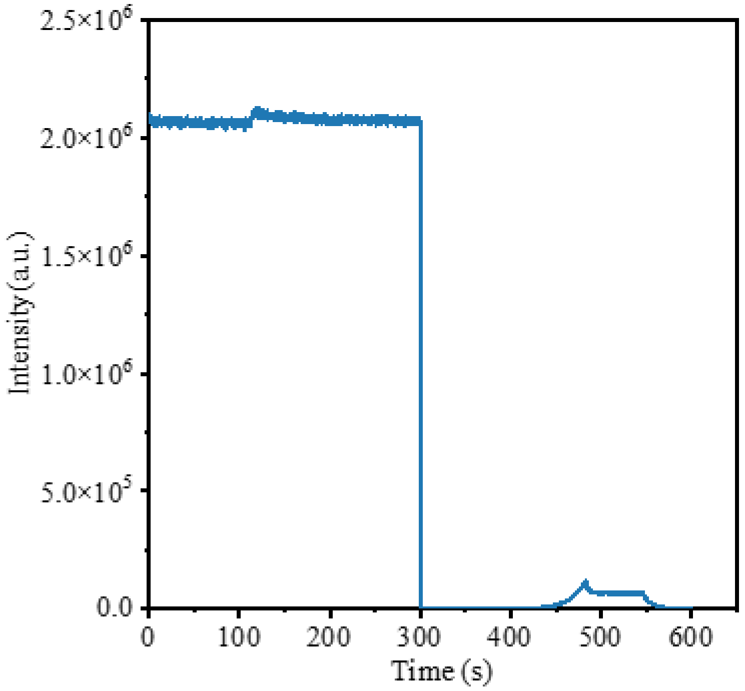

Figure S15. Thermoluminescence spectrum of the $K_2Sr_{0.88}Al_{0.1}P_2O_7$:0.02$Cr^{3+}$ phosphor.

The thermoluminescence spectrum was obtained by initially irradiating the sample at 450 nm for 300 s. After irradiation, the light source was turned off, and the sample was heated at a rate of 423 K/min until the temperature reached 773 K, as shown in Figure S15. The temperature was then held at 773 K for 60 s. The peak observed at 480 s may correspond to black-body radiation.

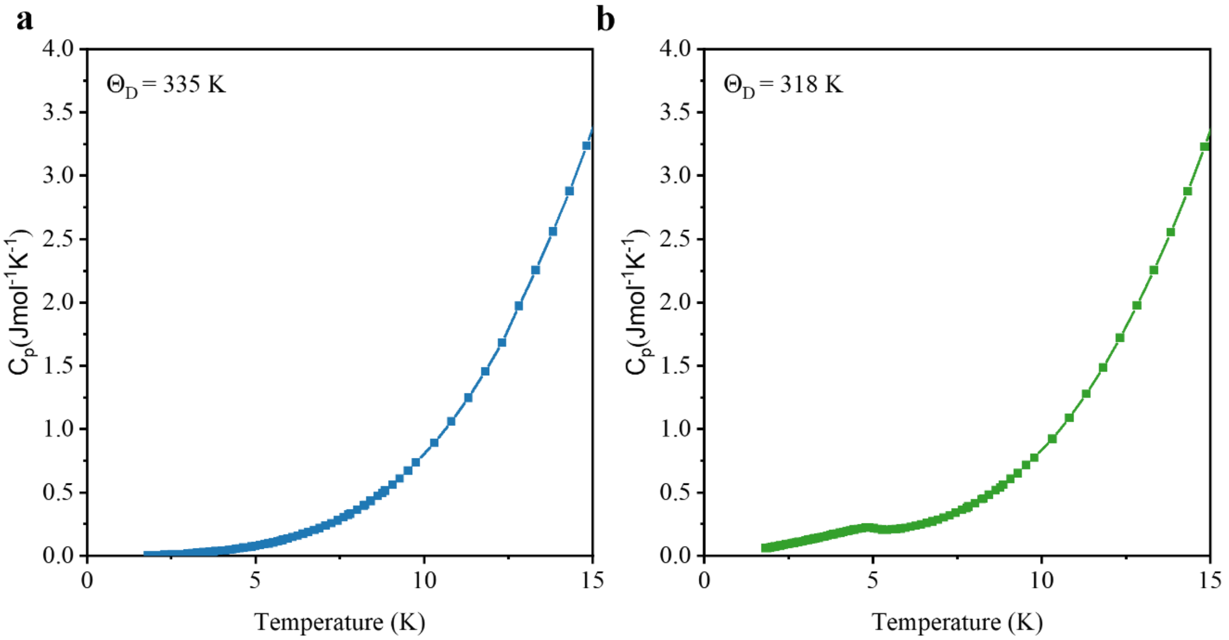

Figure S16. Low temperature specific heats and extracted Debye temperature of the (a) $K_2SrP_2O_7$ and (b) $K_2Sr_{0.9}Al_{0.1}P_2O_7$ host compounds.

The Debye temperature (Θ_D) were extracted from the data fittings of the specific heat in 2-15 K range to the Debye model in the low temperature limit:[13]

$$C_p \approx \frac{12Nk_B\pi^4}{5}\left(\frac{T}{\Theta D}\right)^3 \tag{S8}$$

where N is the number of atoms per formula unit multiplied by the Avogadro number, k_B is the Boltzmann constant, and T is the temperature.

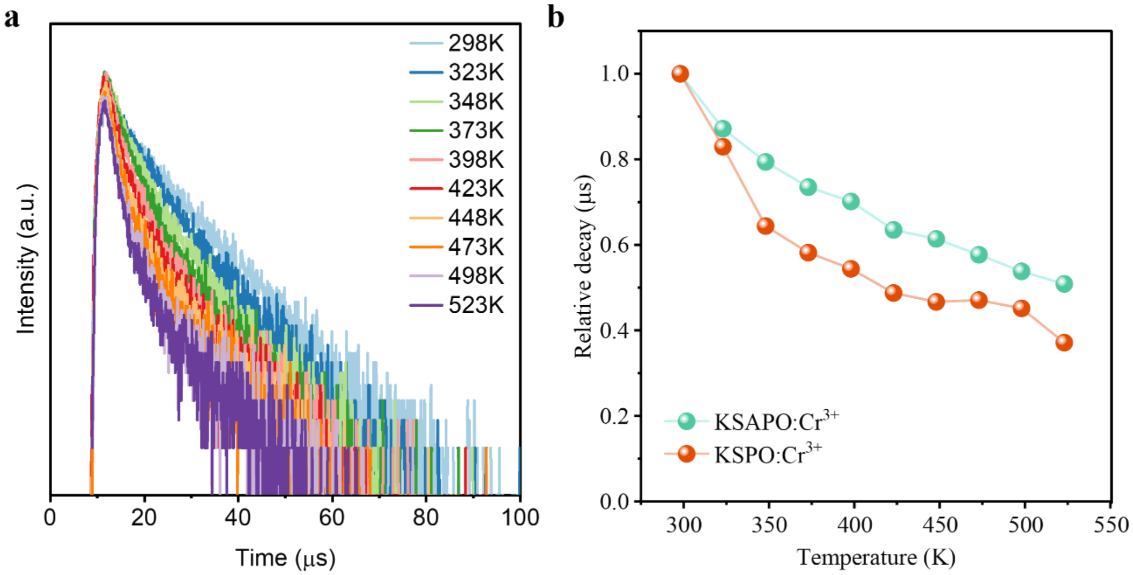

Figure S17. (a) PL decay curves of the $K_2Sr_{0.98}P_2O_7$:0.02Cr^{3+} phosphor at different temperatures. (b) Relative decay time of $K_2Sr_{0.98}P_2O_7$:0.02Cr^{3+} (labeled as KSPO:Cr^{3+}) and $K_2Sr_{0.88}Al_{0.1}P_2O_7$:0.02$Cr^{3+}$ (labeled as KSAPO:Cr^{3+}) phosphors with different temperatures.

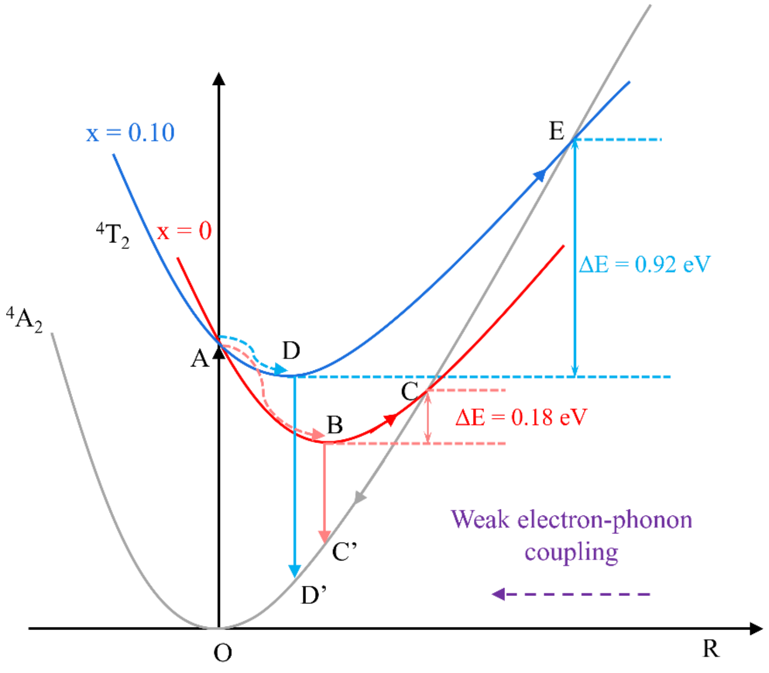

Figure S18. Configuration coordinate curves illustrating the thermal quenching of Cr^{3+} luminescence.

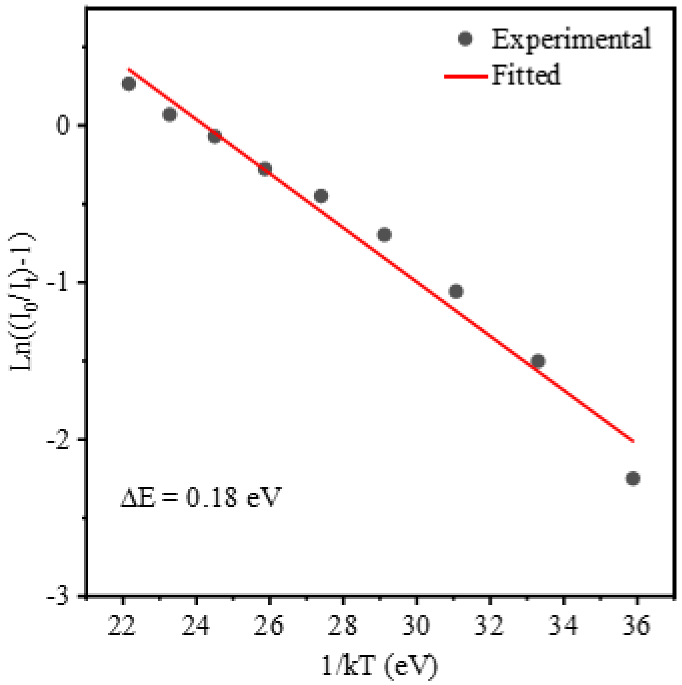

Figure S19. Plot of relationship between ln(I_0/I_T - 1) versus of 1/kT of the $K_2Sr_{0.98}P_2O_7$:0.02Cr^{3+} phosphor.

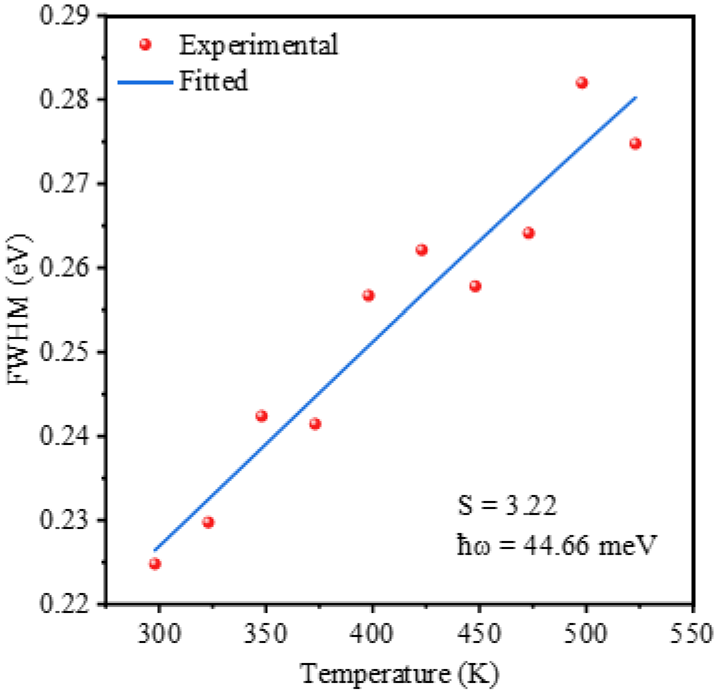

Figure S20. Plot of the temperature dependence of the FWHM of the $K_2Sr_{0.98}P_2O_7$:0.02Cr^{3+} phosphor.

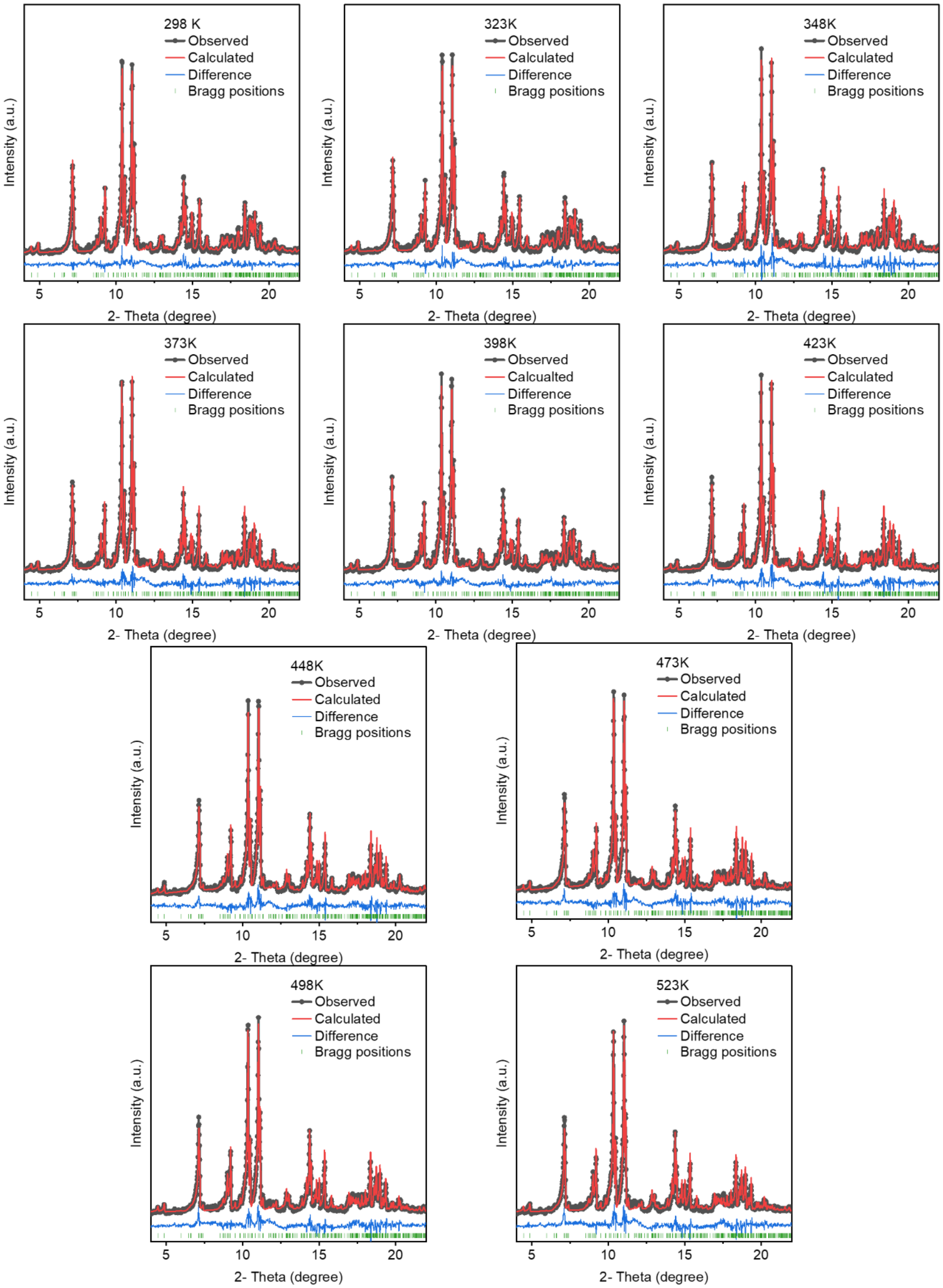

Figure S21. Rietveld refinement of the $K_2Sr_{0.88}Al_{0.1}P_2O_7$:$0.02Cr^{3+}$ phosphor at different temperatures.

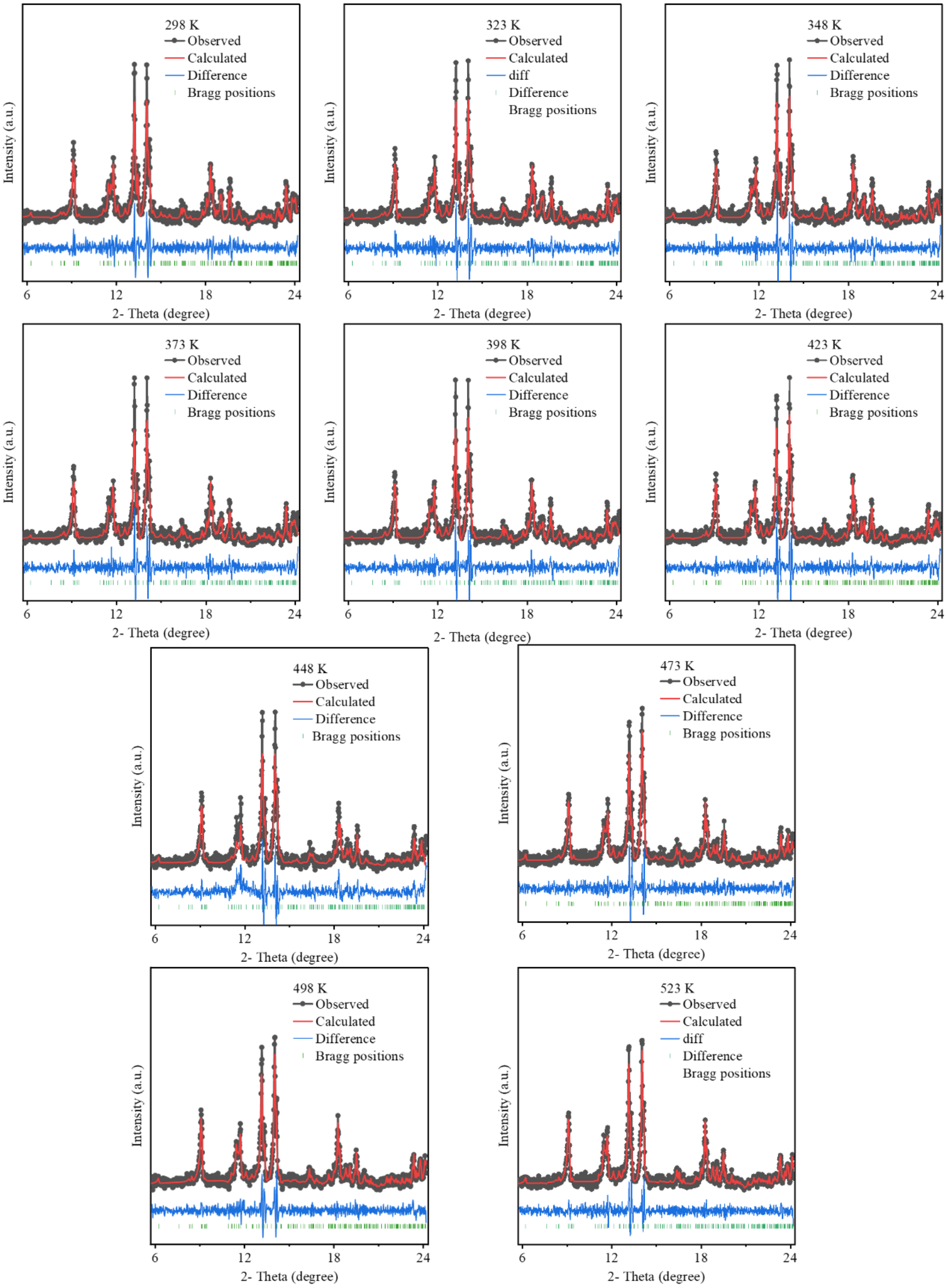

Figure S22. Rietveld refinement of the $K_2Sr_{0.98}P_2O_7$:0.02Cr^{3+} phosphor at different temperatures.

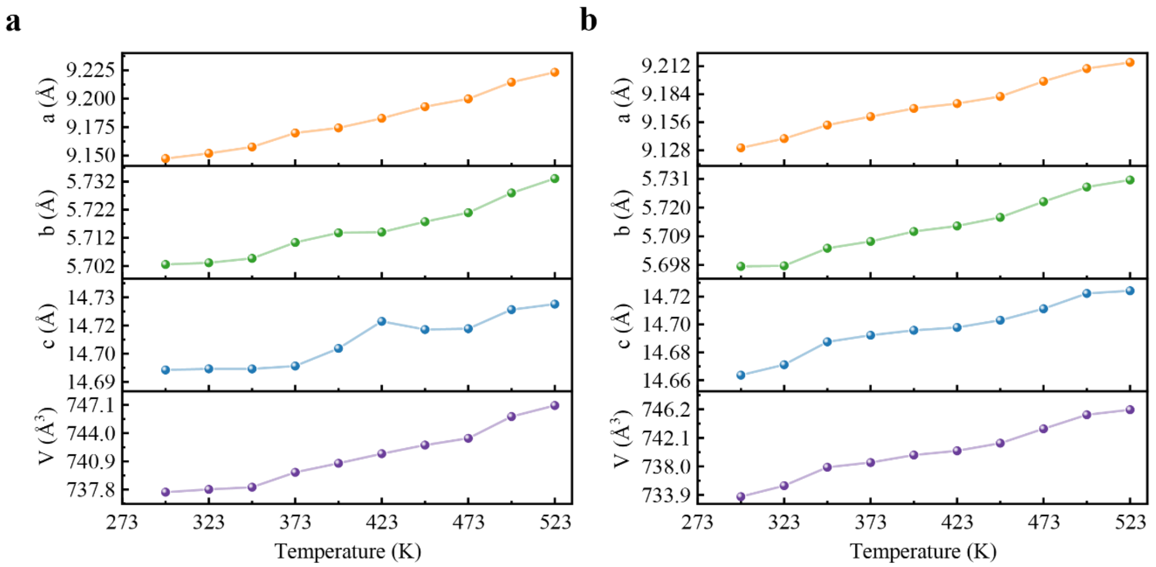

Figure S23. Refined lattice parameters of *a*, *b*, *c*, and *V* of the (a) $K_2Sr_{0.98}P_2O_7$:0.02Cr^{3+} and (b) $K_2Sr_{0.88}Al_{0.1}P_2O_7$:0.02$Cr^{3+}$ phosphors at different temperatures.

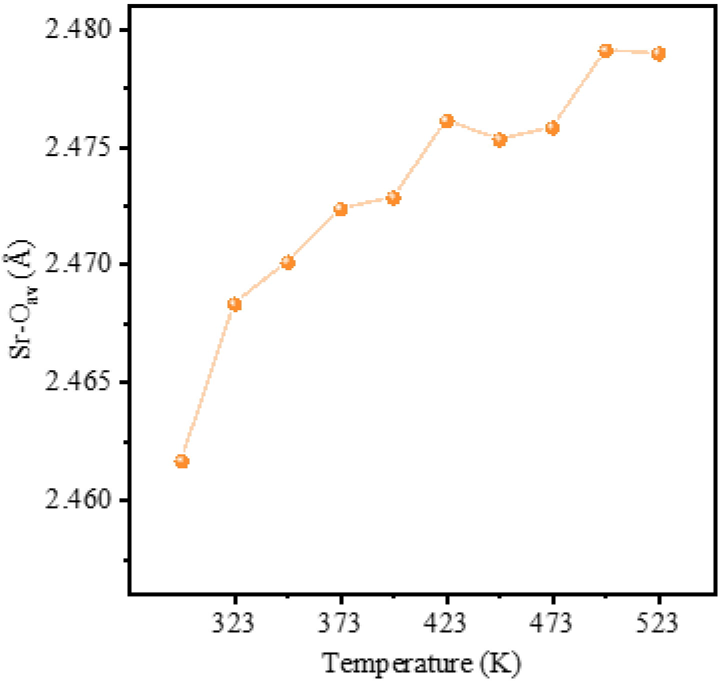

Figure S24. The average Sr-O bond length as a function of temperature of the $K_2Sr_{0.98}P_2O_7$:0.02Cr^{3+} phosphor measured in the temperature range of 298 – 523K.

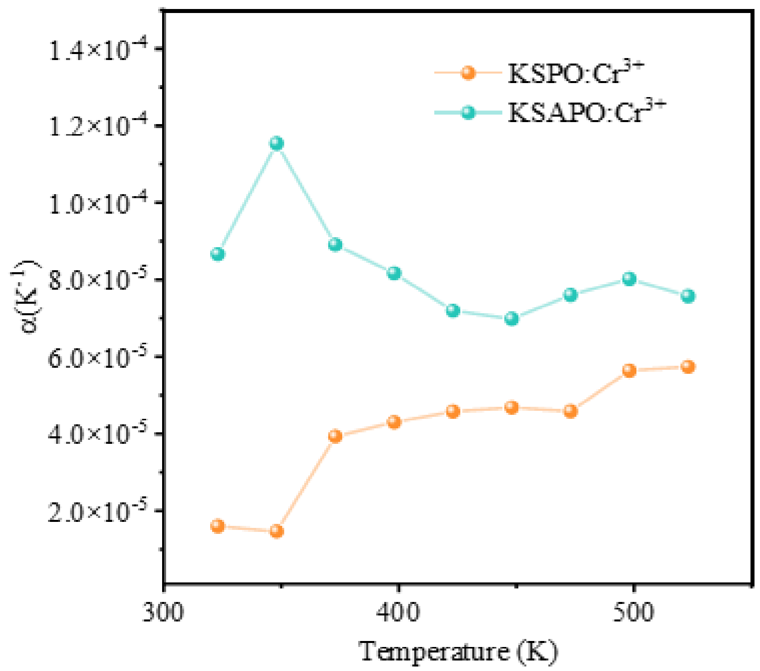

Figure S25. Related thermal expansion coefficient calculation of $K_2Sr_{0.98}P_2O_7$:0.02Cr^{3+} (labeled as KSPO:Cr^{3+}) and $K_2Sr_{0.88}Al_{0.1}P_2O_7$:0.02$Cr^{3+}$ (labeled as KSAPO:Cr^{3+}) phosphors with varied temperatures.

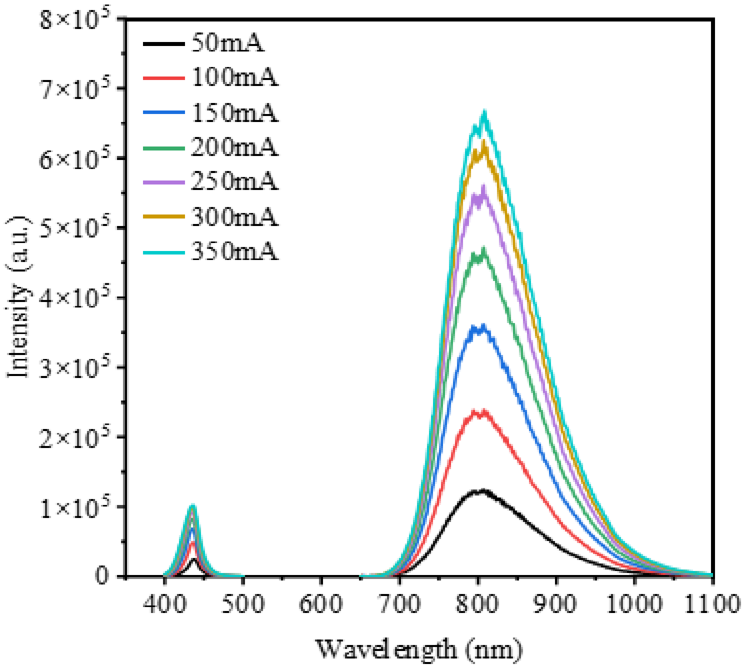

Figure S26. Electroluminescence spectra of the prepared NIR pc-LED using the $K_2Sr_{0.88}Al_{0.1}P_2O_7$:0.02$Cr^{3+}$ phosphor at elevated driven currents.

References:

1. Jiang, C.; Yang, K.; Li, K.; Zhang, D.; Li, Y.; Fu, Y.; Feng, Y.; Zhang, H.; Wei, B.; Liu, Q., Hetero-valent substitution induced luminescence enhancement of $Zn_{1-x}Ga_{2+x}O_{4+\delta}:Cr^{3+}$ near infrared phosphors for plant cultivation. *J. Lumin.* **2023,** *263*, 120140.
2. Dang, P.; Zhang, Q.; Liu, D.; Li, G.; Lian, H.; Shang, M.; Lin, J., Hetero-valent substitution strategy toward orange-red luminescence in Bi^{3+} doped layered perovskite oxide phosphors for high color rendering index white light-emitting diodes. *Chem. Eng. J.* **2021,** *420*, 127640.
3. Chang, H.; Xiao, Z.; Liu, J.; Jiang, W.; Gou, J.; Guo, X.; Qin, S.; Zhang, Q.; You, W.; Han, L., Dopant and compositional modulation triggered long-wavelength ultra-broadband and tunable NIR emission in $MgO:Cr^{3+}$ phosphor for NIR spectroscopy applications. *Ceram. Int.* **2023,** *49* (1), 309-322.
4. Cheng, C.; Ning, L.; Ke, X.; Molokeev, M. S.; Wang, Z.; Zhou, G.; Chuang, Y.-C.; Xia, Z., Designing High-Performance LED Phosphors by Controlling the Phase Stability via a Heterovalent Substitution Strategy. *Advanced Optical Materials* **2020,** *8* (2), 1901608.
5. Wang, J.; Wu, D.; Duan, H.; Liang, H.; Wang, Y.; Peng, J.; Ye, X., Selective Addition of Al^{3+} into $Ba_2SiO_4:Eu^{2+}$ Phosphor to Improve Its Luminescence and Thermal Stability. *ECS Journal of Solid State Science and Technology* **2021,** *10* (6), 066002.
6. Ma, Y.-H.; Gao, X.; Zhang, W.-T.; Yang, Z.-R.; Zhao, Z.; Qu, C., Enhanced red luminescence of $Ca_3Si_{2-x}M_xO_7:Eu^{3+}$ (M = Al, P) phosphors via partial substitution of Si4+ for applications in white light-emitting diodes. *Rare Metals* **2024,** *43* (2), 736-748.
7. Liang, J.; Yang, X.; Xiao, S., Enhancement on the luminescence of $Ca_2B_5O_9Cl:Eu^{2+}$ by co-doping of Al3+ and Ba2+ ions for full spectrum lighting application. *Opt. Mater.* **2023,** *135*, 113269.
8. Liu, S.; Li, L.; Qin, X.; Du, R.; Sun, Y.; Xie, S.; Wang, J.; Molokeev, M. S.; Xi, S.; Bünzli, J.-C. G.; Zhou, L.; Wu, M., Achieving Ultra-Broadband Sunlight-Like Emission in Single-Phase Phosphors: The Interplay of Structure and Luminescence. *Adv. Mater.* **2024,** *36* (38), 2406164.
9. Yu, G.; Liu, H.; Yan, W.; Guo, R.; Wu, A.; Zhao, Z.; Liu, Z.; Bian, Z., 4f → 3d sensitization: a luminescent Eu^{II}–Mn^{II} heteronuclear complex with a near-unity quantum yield. *Materials Horizons* **2023,** *10* (2), 625-631.
10. Fang, M.-H.; Chen, K.-C.; Majewska, N.; Leśniewski, T.; Mahlik, S.; Leniec, G.; Kaczmarek, S. M.; Yang, C.-W.; Lu, K.-M.; Sheu, H.-S.; Liu, R.-S., Hidden Structural Evolution and Bond Valence Control in Near-Infrared Phosphors for Light-Emitting Diodes. *ACS Energy Letters* **2021,** *6* (1), 109-114.
11. Liu, G.; Hu, T.; Molokeev, M. S.; Xia, Z., Li/Na substitution and Yb^{3+} co-doping enabling tunable near-infrared emission in $LiIn_2SbO_6:Cr^{3+}$ phosphors for light-emitting diodes. *iScience* **2021,** *24* (4), 102250.
12. Yu, D.; Zhou, Y.; Ma, C.; Melman, J. H.; Baroudi, K. M.; LaCapra, M.; Riman, R. E., Non-Rare-Earth $Na_3AlF_6:Cr^{3+}$ Phosphors for Far-Red Light-Emitting Diodes. *ACS Applied Electronic Materials* **2019,** *1* (11), 2325-2333.
13. Piespergen, U., Heat capacity and Debye temperatures. In *Semiconductors and semimetals*, Elsevier: 1966; Vol. 2, pp 49-60.
